# Hairy CRISPR: Genome Editing in Plants Using Hairy Root Transformation

**DOI:** 10.3390/plants11010051

**Published:** 2021-12-24

**Authors:** Alexey S. Kiryushkin, Elena L. Ilina, Elizaveta D. Guseva, Katharina Pawlowski, Kirill N. Demchenko

**Affiliations:** 1 Laboratory of Cellular and Molecular Mechanisms of Plant Development, Komarov Botanical Institute, Russian Academy of Sciences, 197376 Saint Petersburg, Russia; eilina@binran.ru (E.L.I.); lguseva@binran.ru (E.D.G.); 2 Department of Ecology, Environment and Plant Sciences, Stockholm University, 10691 Stockholm, Sweden

**Keywords:** *Agrobacterium* strains, hairy root transformation, CRISPR/Cas, genome editing, vector construction, *OsMac3*, pKSEe401R

## Abstract

CRISPR/Cas-mediated genome editing is a powerful tool of plant functional genomics. Hairy root transformation is a rapid and convenient approach for obtaining transgenic roots. When combined, these techniques represent a fast and effective means of studying gene function. In this review, we outline the current state of the art reached by the combination of these approaches over seven years. Additionally, we discuss the origins of different *Agrobacterium rhizogenes* strains that are widely used for hairy root transformation; the components of CRISPR/Cas vectors, such as the promoters that drive Cas or gRNA expression, the types of Cas nuclease, and selectable and screenable markers; and the application of CRISPR/Cas genome editing in hairy roots. The modification of the already known vector pKSE401 with the addition of the rice translational enhancer *OsMac3* and the gene encoding the fluorescent protein DsRed1 is also described.

## 1. Introduction

Interest in the manipulation of genes in the whole plant or its organs has increased since 1983 when several studies first reported the generation of transgenic plants [1]. Hairy root transformation represents a convenient means of studying gene function in plants. This approach is based on the ability of the natural infectious agent, *Agrobacterium rhizogenes* (*Rhizobium rhizogenes*) [2], to infect wounded plants, resulting in the development of numerous roots from the wounded site, known as “hairy root disease”. Although different *A. rhizogenes* strains commonly used for hairy root transformation have been described or reviewed [3,4,5], no report to date has provided a clear description of the relationships among the different *A. rhizogenes* strains. The nomenclature of related strains can vary greatly due to the transfer of *A. rhizogenes* strains between laboratories, leading to the erroneous designation of *A. rhizogenes* strains used in specific studies. Accordingly, a detailed description of *A. rhizogenes* strain nomenclature is provided here, as is the relationship among the different strains.

DNA modification systems such as meganucleases, zinc finger nucleases (ZFNs), transcription activator-like effector nucleases (TALENs), and clustered regularly interspaced short palindromic repeats (CRISPR)-associated (Cas) nucleases are frequently used in studies on plant functional genomics. Meganucleases, ZFNs, and TALENs were the predominantly used DNA modification systems in plant research before the advent of the CRISPR/Cas era [1,6]. In 2013, it was shown that CRISPR/Cas-mediated genome editing could be used to investigate gene function in both plant protoplasts and whole plants [7]. The combination of CRISPR/Cas-mediated genome editing and the hairy root transformation approach was reported one year later [8]. Meanwhile, the number of studies combining these two techniques has grown to 78, involving 26 different plant species.

Here, we outline the general rules applicable to CRISPR/Cas vector construction. Accordingly, a part of this review focuses on the principles of CRISPR/Cas vector design. During the construction of CRISPR/Cas vectors, attention should be paid to the following four parts: which promoters to use to drive the expression of genes encoding different Cas nucleases; which Cas-based system to use; the design and construction of guide RNAs (gRNAs) and the assessment of their efficiency; the choice of genes encoding markers for the identification of transgenic roots.

A variety of constitutive promoters, such as the 35S promoter of the Cauliflower Mosaic Virus (p35S) and its variants (2xp35S, 2xp35SΩ, p35SPPDK), are frequently used to drive *Cas* expression. Other promoters used for CRISPR/Cas-based genome editing include strong promoters, such as pUbi or pActin, as well as inducible or organ- and tissue-specific promoters. The choice of Cas nuclease utilized for genome editing has also varied. Cas nucleases fall into the following three large groups based on their mode of action: those that can introduce double-stranded DNA breaks; those that introduce single-stranded breaks; those that do not introduce breaks. In addition to describing different gene-editing systems, we discuss their use in hairy root transformation, importantly including the choice of marker for the reliable identification of transgenic hairy roots. These markers are commonly divided into two large groups—selectable and screenable (visually) [9]—and we evaluate the advantages and disadvantages of each. New tricks using known markers and several novel markers with potential for future application are also reviewed, as are the research fields where genome editing by hairy root transformation is useful.

## 2. *Agrobacterium rhizogenes*: A Historical View of the Widely Used Strains and Their Nomenclature

The hairy root transformation approach is based on the infection of wounded plants by the naturally existing soil-borne Gram-negative bacterium, *A*. *rhizogenes*. Although more than 20 strains of *A. rhizogenes* (wild-type or generated via genome engineering) [4] and more than 100 plant species are currently available for hairy root transformation [3,5,10], only 14 *A. rhizogenes* strains and 26 plant species have been used in genome editing experiments to date (Table S1). Reviews of *A. rhizogenes* strains are generally restricted to the classification of strains according to the opines they synthesize [4,11]. Here, we have replaced the usual list of *A. rhizogenes* strains with a scheme describing the relationships among them, similar to that recently presented for *A. tumefaciens* strains [12].

**Figure 1:**
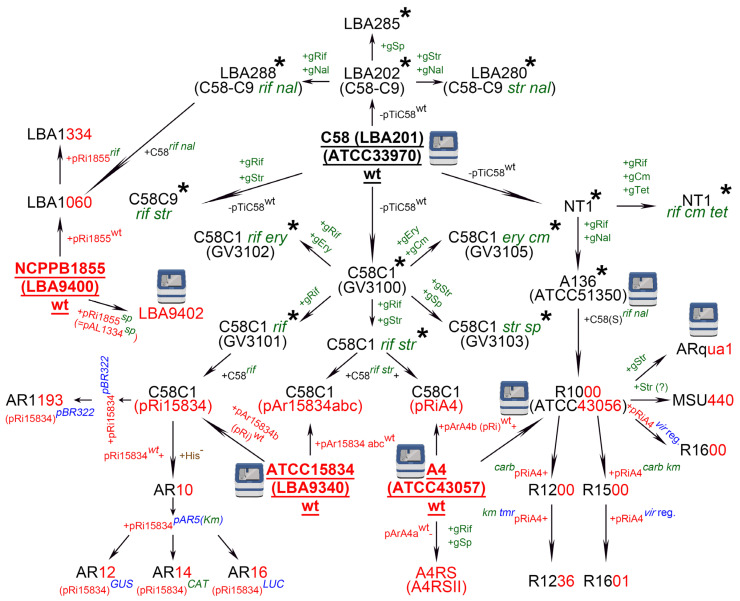
**Relationships between *Agrobacterium rhizogenes* and *Agrobacterium tumefaciens* strains.** *A. rhizogenes* strains are given in red, *A. tumefaciens* strains in black, transconjugants between *A. rhizogenes* and *A. tumefaciens* are given in both colors. The abbreviations of the four main ancestral wild type *Agrobacterium* strains are highlighted by bold print and underlining: A4, ATCC15834, NCPPB1855, C58. Arrows show the directions of isolation of derivaties. Parts of *Agrobacterium* strain abbreviations: AR—*Agrobacterium rhizogenes*; ARqua1—*Agrobacterium rhizogenes* strain obtained by H.J. Quandt; ATCC—American Type Culture Collection; A4RS—A4 derivative resistant to rifampicin and spectinomycin; C58—*A. tumefaciens* strain isolated from cherry gall in 1958; C58C1 or C58C9—C58 strain cured of Ti plasmid (pTi), the numeric designation 1 or 9 refers to a colony number; GV—Ghent University and Vrije Universiteit Brussel; LBA—*Lugdunum Batavorum Agrobacterium*; MSU—Michigan State University; NCPPB—National Collection of Plant Pathogenic Bacteria. Plus or minus indicates a transfer or loss of any component in the resulting *Agrobacterium* strain. The chromosomal background or the Ri plasmid transferred to a new *A. rhizogenes* strain are indicated next to the arrows in black or red, respectively. Antibiotic resistances or other modifications of the chromosomal background or of the Ri plasmids are designated as follows. C58 derivatives cured of the Ti plasmid are indicated with an asterisk. WT stands for wild type *Agrobacterium* strain or for its native plasmids (pTi or pRi). Antibiotic resistances appearing either in the chromosome (g) or as inserts in pRi are given in green; Carb—carbenicillin, Cm (CAT)—chloramphenicol, Ery—erythromycin, Km—kanamycin, Nal—nalidixic acid, Sp—spectinomycin, Str—streptomycin, Tet—tetracycline; a question mark denotes suggested resistance. Other modifications inserted into pRi (given in blue): pAR5—vector harboring genes encoding either *β*-glucuronidase (GUS) or chloramphenicol acetyltransferase (CAT) or luciferase (LUC), was integrated into wild type pRi15834 of the strain AR10 *his*^−^ via homologous recombination; pBR322—part of the pBR322 sequence inserted into wild type pRi15834 of the strain C58C1 (pRi15834) via homologous recombination; *tmr*—cytokinin synthesis locus encoding an isopentenyl transferase; *vir* reg.—part of the *vir* region from pTiBO342 (conferring the supervirulent phenotype to *A. tumefaciens* A348). Other modifications (given in brown): His^-^—histidine auxotrophy acquired by random mutagenesis of C58C1 (pRi15834). The sequencer icon (created with BioRender) indicates *Agrobacterium* strains with sequenced genome.

The 15,834 strain from the American Type Culture Collection (ATCC—also known as ATCC15834, 15834, or AR15834) [13] (Figure 1) was one of the first isolated wild-type *A. rhizogenes* strains to be widely used for hairy root transformation. The strain was mentioned for the first time in the 1980s by Frank F. White and Eugene Nester while investigating the virulence of root-inducing (Ri) and tumor-inducing (Ti) plasmids (pRi and pTi) [14,15]; however, ATCC15834 was likely isolated earlier, probably in 1971. ATCC15834 was deposited at the ATCC by Richard D. Durbin, who had obtained the strain from Peter A. Ark [13]. Durbin described a highly virulent *A. rhizogenes* strain that was originally isolated from rose by P.A. Ark [16] and we assume that this was the strain that was later designated as ATCC15834. The ATCC15834 strain harbors three large plasmids, namely, pAr15834a, b, and c (also designated as pAr15834abc) [14] (Figure 1). Because pAr15834b represents the pRi that is responsible for hairy root induction, it is often designated as pRi15834 [14] (Figure 1). The draft genome sequence of ATCC15834 was published for the first time in 2014, based on Illumina sequencing [17], and then again in 2020 based on combined Illumina and Oxford Nanopore sequencing [13] (Figure 1).

Another wild-type *A. rhizogenes* strain is A4, also designated as ATCC43057 [18] (Figure 1). Presumably, similarly to ATCC15834, this strain was isolated by P.A. Ark from naturally infected roses exhibiting a hairy root phenotype [19]. Again, as with ATCC15834, the A4 strain contains three large plasmids, pArA4a, b, and c, one of which, pArA4b, also called pRiA4, is responsible for hairy root induction [20] (Figure 1). The A4 draft genome was published first in November 2020 by the ATCC based on a combination of Oxford Nanopore with Illumina sequencing [18] and then independently in May 2021 by a research group from the Academia Sinica (Taipei, Taiwan) [21] (Figure 1). In 1987, Frank F. White proposed that A4 might be identical to strain ATCC15834 [22]; however, this possibility has not been tested to date. A derivative of A4 that is resistant to rifampicin and spectinomycin (named A4RS, based on the initials of the antibiotics) and lacks pArA4a [20] (Figure 1) is frequently used for hairy root transformation [23,24,25]. Some studies have used a slightly different designation for this strain, i.e., A4RSII [26,27] (Figure 1).

Two wild-type *A. rhizogenes* strains from the National Collection of Plant Pathogenic Bacteria (NCPPB), NCPPB2659 (commonly known as K599, not included in Figure 1) [28] and NCPPB1855 [29] (Figure 1), also deserve mention. K599 was isolated from cucumber exhibiting hairy root disease symptoms [30] and has been widely used for hairy root transformation in legumes such as *Arachis hypogea*, *Glycine max*, *Glycine soja*, and *Vigna unguiculata* (Table S1). Although pRi from K599 was sequenced in 2007 [31], the draft genome of this strain was only published much later, first in 2016 [32], and then in 2021 [33]. Strain NCPPB1855 originates from *Rosa* sp. [34] and is also known as LBA9400 [35] (Figure 1). This strain gave rise to a rifampicin-resistant derivative, LBA9402 [35], the genome of which was sequenced in 2021. This was the first fully assembled *A. rhizogenes* genome [34] (Figure 1). The abbreviation LBA stands for *Lugdunum Batavorum Agrobacterium* [36]. *Lugdunum Batavorum* is the (erroneous) Latin name for Leiden in the Netherlands. *Agrobacterium* strains with names beginning with LBA were either isolated at Leiden University or transferred to its strain collection from other laboratories. For instance, ATCC15834 has the designation LBA9340 in the Leiden collection [35] (Figure 1).

Several *A. rhizogenes* strains currently used for hairy root transformation [4,5] are transconjugants. To understand the relationships between transconjugant and wild-type *A. rhizogenes* strains, we partially review the history of different *A. rhizogenes* strains here. Many transconjugant *A. rhizogenes* strains are closely related to the *A. tumefaciens* strain C58 and have the C58 chromosomal background along with a pRi from the wild-type *A. rhizogenes* strains described above (Figure 1). Accordingly, understanding the history of *A. rhizogenes* strains also requires a review of the data on *A. tumefaciens* strains. *A. tumefaciens* strain C58 was originally isolated in 1958 by Robert Dickey (Cornell University, Ithaca, NY, USA) from a cherry gall (C designates cherry and 58 the year of collection). The draft C58 genome was published twice independently in 2001 [37,38] (Figure 1). A comparison of these sequencing data in 2013 showed that the C58 strain originating in Eugene Nester’s laboratory (Washington University, Seattle, WA, USA) was present as two isolates [39]. The first sequenced isolate was the C58 strain stored in the laboratory of Eugene Nester at the University of Washington. The second sequenced isolate also originated from Nester’s laboratory and was deposited at the ATCC in 1981 (ATCC33970) (Figure 1), subcultured three times by the ATCC and once at Monsanto Company, and then sequenced in 2001. The genomes of the two compared C58 isolates showed 30 true differences, including two deletions [39].

The history of the transconjugant *A. rhizogenes* strains began in the 1970s when it was found that *A. tumefaciens* C58 lost its pTi plasmid when grown at 37 °C [40,41], leading to the generation of *A. tumefaciens* strains cured of pTi. Three research groups (one in the USA, one in Belgium, and one in the Netherlands) independently isolated several C58 strains lacking pTi. Eugene Nester’s group (USA) cured the C58 strain (obtained from R. H. Hamilton at Pennsylvania State University) of its pTi using the method of Hamilton and Fall [40] and named the resulting strain NT1 [42] (Figure 1). In the same study, strain A136, which is resistant to rifampicin and nalidixic acid, was isolated from NT1. This strain was later deposited at the ATCC under the number 51350 and its genome sequence was published in 2020 [43]. There is also a derivative of NT1 that is resistant to rifampicin, chloramphenicol, and tetracycline (NT1 *rif cm tet*) [19] (Figure 1).

A series of C58 strains cured of the pTi was generated in a collaboration between Ghent University, the Vrije Universiteit Brussel (both in Belgium), and the University of Leiden (Netherlands) [12,41,44]. A C58 strain originally obtained from Milton P. Gordon (University of Washington) [41] gave rise to several cured strains (Figure 1), one of which was named C58C1 [44] (with C indicating that it was cured of the pTi) [12] (Figure 1). There are at least five avirulent derivatives of C58C1, each with a different antibiotic resistance profile, namely, resistance to rifampicin and streptomycin (C58C1 *rif str*) [44]; rifampicin and erythromycin (C58C1 *rif ery*) [45]; streptomycin and spectinomycin (C58C1 *str sp*) [46]; rifampicin (C58C1 *rif*); erythromycin and chloramphenicol (C58C1 *ery cm*) [47] (Figure 1). In subsequent studies, C58C1 and its antibiotic-resistant derivatives were given new designations beginning with the letters GV (standing for Ghent University and the Vrije Universiteit Brussel [12]), as follows: C58C1, without any antibiotic resistance, was designated GV3100 [47,48]; C58C1 *rif* was designated GV3101 [47]; C58C1 *rif ery* was designated GV3102 [46,47]; C58C1 *str sp* was designated GV3103 [46,47]; and C58C1 *ery cm* was designated GV3105 [47] (Figure 1). However, we did not find a GV designation for C58C1 *rif str* from the study of Larebeke et al. [44], and thus this strain does not have a GV name in our scheme (Figure 1). An equivalent of C58C1 *rif str* described by Larebeke et al. in 1975 [44] was another strain, C58C9, newly isolated by Tempé et al. two years after Larebeke’s study, in 1977 [49]. Because antibiotic resistances of C58C9 are the same as those of C58C1 *rif str* [44], we designated this strain C58C9 *rif str* (Figure 1).

Another *A. tumefaciens* strain, C58-C9, was isolated by the group of Jeff Schell (Leiden University, Netherlands) [50,51] in a manner analogous to that of C58C1 (GV3100). C58-C9, which is cured of the pTi, originated from wild-type *A. tumefaciens* C58 [41], and was renamed LBA201 according to Leiden University nomenclature [52] (Figure 1). Given that the chromosomal background of C58-C9 was shown to be characteristic of that of *A. tumefaciens* strain LBA202 [53], and that LBA202 represents C58 cured of its pTi [36,54], we propose that C58-C9 and LBA202 are synonyms for the same *A. tumefaciens* strain which is designated as LBA202 (C58-C9) in Figure 1. Three spontaneous derivatives of LBA202 (C58-C9) were subsequently obtained. One was resistant to nalidixic acid and streptomycin (LBA280 or C58-C9 *str nal*); one to nalidixic acid and rifampicin (LBA288 or C58-C9 *rif nal*) [53,54]; and one to spectinomycin (LBA285) [53] (Figure 1).

Somewhat confusingly, Tempé et al. reported the isolation of a C58C9 strain [49]. The C58-C9 strain from Leiden University has no antibiotic resistance [50,51,53], whereas C58C9 from Tempé et al. is resistant to rifampicin and streptomycin [49] (Figure 1). C58-C9 without antibiotic resistance was reported by both Bomhoff et al. [50] and Ledeboer et al. [51] in 1976, while C58C9 *rif str* was reported by Tempé et al. in 1977 [49]. Therefore, the C58C9 *rif str* strain isolated by Tempé et al. [49] could have been a derivative of either LBA202 (C58-C9) or the original wild-type *A. tumefaciens* C58 [41]. In our scheme, we have placed the C58C9 *rif str* strain independently from LBA202 (C58-C9) based on the data provided by Tempé et al. [49] (Figure 1).

As both LBA288 (C58-C9 *rif nal*) from the Leiden collection [53,54] and A136 from the ATCC (ATCC51350) [43] are derivatives of C58 cured of the pTi, and both are resistant to rifampicin and nalidixic acid, they should have the same chromosomal background (Figure 1). However, the sequence of A136 with the chromosomal background of C58 [also named Seattle C58, C58(S)] is slightly different from that of the previously sequenced “Ghent/Leiden C58C” chromosome of nopaline catabolizing, plasmid-cured *Agrobacterium* strains [55].

*A. tumefaciens* strain A136, with resistance to rifampicin and nalidixic acid in the C58(S) chromosomal background, and the wild-type *A. rhizogenes* strain A4 gave rise to *A. rhizogenes* R1000 [56] (Figure 1). Some studies state that the R1000 pRi is derived from strain A4T [56,57,58,59,60]; however, we did not find any studies describing the isolation of strain A4T. In a personal communication, Frank F. White (University of Florida, Gainesville, Florida, USA) also confirmed that R1000 has the C58(S) background from A136 and the pRi from A4, not A4T (Figure 1). Nevertheless, in several studies, A4T appears as a separate strain used for hairy root transformation [61,62]. Thus, it remains unclear whether A4 and A4T are the same or separate strains (T in A4T might merely indicate that A4 is a type strain), and a comparative analysis of the genome and plasmid sequences of A4, A4T, A136, and R1000 may be required to resolve the uncertainty with this nomenclature. R1000 is also known under its strain collection name ATCC43056 [63] (Figure 1), and in one study as 43056 [64]. The R1000 genome was published by ATCC in April 2021 [63], with the results showing a 96.62% average nucleotide identity (ANI) between the genomes of R1000 (ATCC43056) and C58 (ATCC33970) and 98.48% ANI between the genomes of R1000 and A136 (ATCC51350) [65]. These findings show that R1000 has the *A. tumefaciens* chromosomal background.

Six strains—R1200, R1236, R1500, R1600, ARqua1, and MSU440—are derived from R1000 (Figure 1). R1200 was obtained by the insertion of a Tn*3* transposon conveying carbenicillin resistance (CarbR) into the *rol* region of R1000 pRiA4 [57] (Figure 1). The cytokinin synthesis locus *tmr* from pTiA6NC, which encodes an isopentenyl transferase, together with Tn*5*, which carries a kanamycin resistance (KmR) gene, were subsequently inserted into pRiA4 of R1200 via homologous recombination, yielding R1236 that carries a CarbR gene, a KmR gene, and the *tmr* locus [57] (Figure 1). R1200 has been widely used for hairy root induction in different species, including belladonna (*Atropa belladonna*) [66], maroon cucumber (*Cucumis anguria*) [67], and Tartary buckwheat (*Fagopyrum tataricum*) [68].

R1500 was generated by the insertion of a chimeric KmR gene into a defective Tn*3* transposon (pET23) carrying the CarbR gene, followed by the insertion of the Tn*3* transposon into pRiA4 via homologous recombination [69] (Figure 1). R1600 and R1601 were constructed via the modification of, respectively, the R1000 and R1500 strains with the pTVK291 cosmid harboring part of the *vir* region of pTiBO342 (conferring the supervirulent phenotype of *A. tumefaciens* A348) [70] (Figure 1). ARqua1 is a streptomycin-resistant derivative of R1000 [71] (Figure 1). The abbreviation ‘AR’ in the ARqua1 strain stands for *A. rhizogenes* and ‘qua’ for its creator, Hans Joachim Quandt (Inge Broer, personal communication). ARqua1 is also resistant to spectinomycin, because resistance to streptomycin normally provides cross-resistance to spectinomycin (our data, unpublished). The genome of ARqua1 with pRiA4 was sequenced in 2020 [60] (Figure 1).

The *A*. *rhizogenes* MSU440 strain was first mentioned in a study by Sonti et al. [72], where the authors stated that the MSU440 strain was obtained from Chris R. Somerville and contains the pRi from the A4 strain [72]. The abbreviation ‘MSU’ stands for the Michigan State University (Chris R. Somerville, personal communication). The origin of the MSU440 chromosomal background was not mentioned in Sonti et al. MSU440 was derived from R1000 and, therefore, has the pRi from the *A. rhizogenes* A4 strain and the C58 chromosomal background from the *A. tumefaciens* A136 strain (Chris R. Somerville, personal communication) (Figure 1). MSU440 may also be resistant to streptomycin [73] (Figure 1).

The chromosomal background of *A. tumefaciens* C58C1 *rif str* together with the pRi from *A. rhizogenes* A4 or pAr15834abc from ATCC15834 gave rise to, respectively, the C58C1 (pRiA4) and C58C1 (pAr15834abc) strains [74] (Figure 1). Besides C58C1 (pAr15834abc), a rifampicin-resistant C58C1 strain containing only pAr15834b (pRi15834) also exists [75,76,77], which we have designated C58C1 (pRi15834) (Figure 1) based on the fact that two separate strains—C58C1 (pAr15834abc) *rif str* [74] and C58C1 (pRi15834) *rif* [75,76,77]—are described in the literature. However, we did not find any studies describing a transconjugation between the chromosomal background of C58C1 *rif* (GV3101) and the pRi from strain ATCC15834.

Similarly to De Saeger et al. [12], who highlighted that the nomenclature of some published *A. tumefaciens* strains is frequently confusing, incomplete, or misleading, we note with regret the same fact about *A. rhizogenes* strains that have the *A. tumefaciens* C58C1 chromosomal background. For instance, in some studies on hairy root transformation, the *A. rhizogenes* strain used was indicated only by its chromosomal background C58C1 (see Table S1 [63,69,73]). We believe that it would be more appropriate to also indicate the pRi that was introduced into the *A. tumefaciens* strain. Good examples are transconjugant *A. rhizogenes* strain names such as C58 (pRiARqua1) and C58C1 (pRiA4) (see Table S1 [6,70,71]).

C58C1 (pRi15834) *rif* gave rise to several derivatives, including AR1193 [77], AR10, AR12, AR14, and AR16 [75]. The letters “AR” in these strain names presumably also stand for “*A. rhizogenes”*. AR1193 was obtained by the integration of a fragment from pBR322 [78] into the wild-type pRi15834 TL-DNA from strain C58C1 (pRi15834) *rif*, thus forming the basis for further insertion of target genes into the pRi by homologous recombination [77] (Figure 1). The name pBR322 follows the standard rules of plasmid nomenclature, i.e., the “p” stands for plasmid and the “BR” for the names of its creators Francisco Bolivar and Raymond L. Rodriguez, while the numeric designation, 322, stands for the number of the vector in the collection [79]. Strain AR10 represents a histidine auxotrophic mutant (*his*^−^) of C58C1 (pRi15834) *rif* [75] (Figure 1). This mutant was isolated to improve the counterselection of *A. rhizogenes* during the removal of the strain from plants after inoculation. Normally, antibiotics such as cefotaxime, augmentin, and timentin are used to decontaminate plant tissue [80,81]. The histidine auxotrophic derivative of *A. rhizogenes* AR10 can be easily removed as it cannot survive in medium without histidine [75]. AR10 *his*^−^ gave rise to the strains AR12, AR14, and AR16 [75] (Figure 1), which were obtained via homologous recombination between the wild-type pRi15834 plasmid of AR10 and the pBR322-based plasmid, pAR5, harboring genes encoding *β*-glucuronidase (GUS), chloramphenicol acetyltransferase (CAT), or luciferase (LUC), respectively (Figure 1). Thus, the AR12, AR14, and AR16 strains have modified T-DNA containing selectable (CAT) or screenable (GUS or LUC) marker genes that can be used for the identification of transgenic hairy roots [75]. Moreover, part of the pBR322 sequence that is transferred from pAR5 to pRi15834 can form the basis for further insertion of another target gene into pRi15834 by homologous recombination [82].

The transconjugant *A. rhizogenes* LBA1334 strain has the C58C9 chromosomal background with rifampicin resistance [83] and the pRi from NCPPB1855 that carries a spectinomycin resistance gene [83,84] (Figure 1). At least two *A. tumefaciens* strains, C58C9 *rif str* [49] and LBA288 (C58-C9 *rif nal*) [53,54], have the C58C9 background with rifampicin resistance (Figure 1), rendering it problematic to identify the original *A. tumefaciens* strain that provided the chromosomal background for the *A. rhizogenes* LBA1334 strain. LBA1334 was obtained in two steps (Paul J. J. Hooykaas, personal communication). First, *A. rhizogenes* strain LBA1060 was obtained via the transfer of the pRi of *A. rhizogenes* NCPPB1855 into the chromosomal background of the *A. tumefaciens* strain LBA288 (C58-C9 *rif nal*) [53,54] (not that of the *A. tumefaciens* C58C9 *rif str* strain obtained by Tempé et al. [49]) (Figure 1). In the second step, the pRi of NCPPB1855 in LBA1060 was modified by adding a spectinomycin resistance gene (the pRi was named pAL1334) [83,84] (Figure 1). The AL in the pAL1334 stands for *Agrobacterium* Leiden. Strain LBA1334 also carries a nalidixic acid resistance gene from the C58C9 chromosomal background of LBA288 (C58-C9 *rif nal*) [53,54] (Figure 1). LBA1334 might also display chloramphenicol resistance [85] (Figure 1) given that resistance to nalidixic acid normally provides cross-resistance to chloramphenicol (Paul J. J. Hooykaas, personal communication).

The *A. rhizogenes* strains described above have various levels of virulence reflected by their ability to induce hairy root formation in different plant species [3]. One way to increase hairy root transformation efficiency is to change the virulence of *A. rhizogenes* strains, as has been done for the R1600 and R1601 strains [70]. The initial strains, R1000 and R1500, from which R1600 and R1601 were isolated (Figure 1), could not induce hairy roots on hybrid poplar (*Populus trichocarpa* × *deltoides*). Enhancing the virulence of R1000 and R1500 by adding the pTVK291 cosmid that carries part of the *vir* region of pTiBO342 (conferring the supervirulent phenotype to the *A. tumefaciens* strain A348) permitted the generation of hybrid poplar hairy roots and the subsequent regeneration of shoots from those hairy roots [70]. A similar modification was carried out for *A. tumefaciens*, i.e., the stable transformation rate of tomato (*Solanum lycopersicum*) with the resulting strain was increased 3.6-fold compared with that for the original GV2260 strain [86].

CRISPR/Cas9-mediated genome editing of existing strains may represent an additional means of obtaining *A. rhizogenes* with higher virulence [4,5]. A prerequisite for successful genome editing of *A. rhizogenes* strains is the availability of a genome sequence, i.e., the sequence of the chromosome and the pRi. To date, the chromosomes and Ri plasmids of several *A. rhizogenes* strains—ATCC15834 [13,17], A4 [18,21], NCPPB2659 (K599) [31,32,33], LBA9402 [34], ARqua1 [60], and R1000 [63]—have been sequenced (Figure 1). CRISPR/Cas9-based editing was performed for several NCPPB2659 (K599) genes, namely *cus,* encoding cucumopine synthase, *rolB*, *rolC,* and *orf13*. Proof of concept that genomes of *Agrobacterium* strains could be successfully edited was obtained in that the induction of hairy root formation on carrot disks by K599 *rolB* and *rolC* mutants was negatively affected [33]. Comparative analysis of the already sequenced genomes of *A. rhizogenes* strains will help to identify the parts that most strongly affect their virulence and thus help to obtain new supervirulent *A. rhizogenes* strains.

## 3. Editing the Plant Genome in Transgenic Hairy Roots: Vector Components

**Figure 2:**
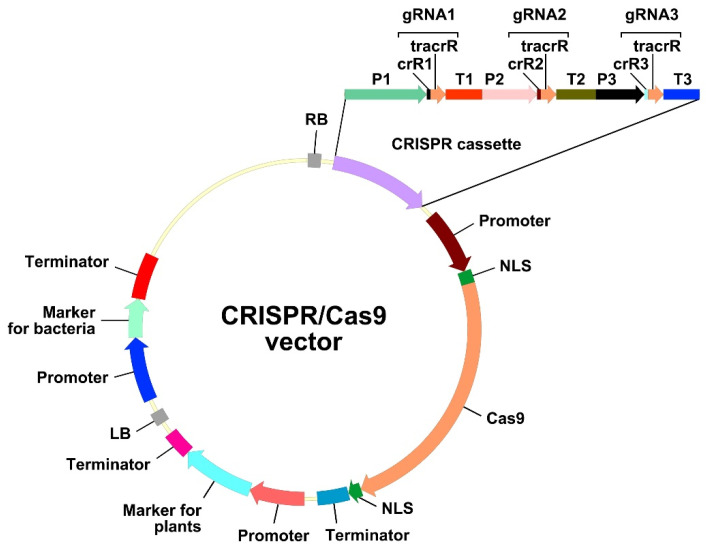
**Map of a CRISPR/Cas9 vector.** Vector components (clockwise): RB—right T-DNA border; CRISPR cassette containing P—promoter of the small nucleolar RNA (*snoRNA*) gene; gRNA—guide RNA; crR—*crisprRNA* (target specific); tracrR—*trans*-activating *crisprRNA* (conserved, used as a binding scaffold for Cas9); T—terminator of the *snoRNA* gene; NLS—nuclear localization signal; Cas9—CRISPR associated nuclease 9; LB—left T-DNA border.

Hairy root transformation has long been used for the modification of plant traits, either because no protocols for stable transformation and regeneration were available, or because the targeted trait was only observed in roots [87]. Another reason for the use of hairy roots is that *A. rhizogenes*-mediated transformation leads to the quick regeneration of the transgenic biomass, which is necessary for the fast production of biomedical/pharmaceutical/industrial molecules of interest [10]. CRISPR/Cas-mediated genome editing can be performed in transgenic hairy roots for the same reasons. A construct for efficient genome editing and selection of hairy roots should contain three components, i.e., a cassette carrying the gene encoding the CRISPR-associated (Cas) nuclease, a cassette expressing the guide RNA (gRNA), and a cassette encoding a screenable or selectable marker (Figure 2).

### 3.1. Cassette for Cas Expression

The construction of a cassette for *Cas* expression in hairy roots depends on the aim of the study. Strong constitutive promoters are typically used to control *Cas* expression in plants. The Cauliflower Mosaic Virus (CaMV) 35S promoter (p35S) [88] is often (in 53 of the 78 studies that have employed CRISPR/Cas in hairy roots) used to drive expression of *Cas9* (Table S1). *Ubiquitin* promoters (pUbi) from different species, including Arabidopsis, parsley (*Petroselinum crispum)*, maize (*Zea mays*), rice (*Oryza sativa*), and soybean (*G. max*), are also popular choices; *Cas9* expression was driven by pUbi in 19 of the 78 studies examined (Table S1). Other strong promoters have been used in individual cases. For instance, the promoter of the Arabidopsis *actin2* gene (pAct2) was used in hairy roots of hybrid poplar (*Populus tremula* × *alba*). In other studies, the 35S enhancer was fused to the maize C4 pyruvate orthophosphate dikinase (C4PPDK) basal promoter (p35SPPDK) [89] to drive *Cas9* expression in hairy roots of rubber dandelion (*Taraxacum kok-saghyz*), while the soybean pSCREAM M4 promoter (pM4), which drives the strong, constitutive expression of elongation factor 1A [90], was employed for *Cas9* expression in soybean hairy roots. Additionally, in one instance, an organ-specific promoter (the promoter of the nodule-specific *leghemoglobin b2* gene of the legume *Lotus japonicus* [pLjLb2]) was used to drive *Cas9* expression in hairy roots of the same species (for pAct2, p35SPPDK, pM4 and pLjLb2 see Table S1 [25,46,52,66,84]).

**Figure 3:**
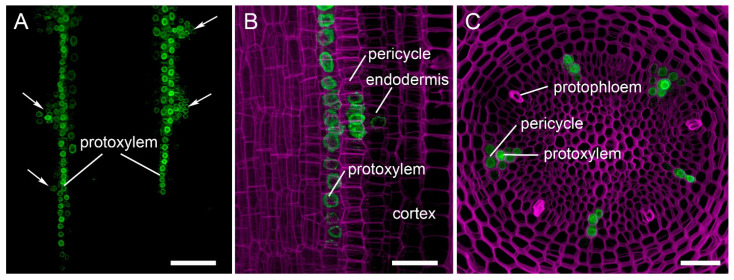
**Localization of *CsRALF34* expression in a *Cucumis sativus* root tip.** Confocal laser scanning microscopy of vibratome longitudinal (**A**,**B**) and cross (**C**) sections of transgenic *pCsRALF34::mNeonGreen-H2B* roots. Green channel—fluorescence of mNeonGreen-H2B; magenta channel—cell walls are counter stained with SCRI Renaissance 2200. (**A**) An overview and (**B**) close-up of the parental root meristem shows the acropetal sequence of *CsRALF34* promoter activity in protoxylem, pericycle and endodermis. *CsRALF34* expression arises first in the protoxylem at a distance of 150 µm from the initial cells. (**C**) The establishment of activity in xylem, pericycle layers and endodermis on a cross section at a distance of 300 µm from the initial cells. Arrows indicate developing lateral root primordia. Scale bars: 100 µm in (**A**), and 40 μm in (**B**,**C**).

Recent advances in plant genome editing have involved the construction of vectors in which tissue-specific [91] or inducible [92] promoters drive *Cas9* expression. Although not all of these genome editing technologies have been applied to hairy roots, they undoubtedly have great potential in the field of root developmental biology. Hairy root transformation, combined with inducible CRISPR/Cas-mediated genome editing, should be used if the target gene has a pleiotropic effect on root development. For instance, the tomato *SHOOT BORNE ROOTLETS* (*SBRL*) gene is involved in the development of both adventitious and lateral roots [93]. In addition to having fewer lateral roots, tomato CRISPR/Cas *sbrl* mutants cannot develop adventitious roots if the main root is removed [93]. Consequently, investigating *SBRL* gene function during lateral root development using hairy root transformation with vectors constitutively expressing *Cas9* will likely not be possible because the edited hairy roots will not develop. CRISPR/Cas-mediated tissue-specific knockout can be used in hairy roots for the same reason, namely, to avoid a pleiotropic effect. Another advantage of tissue-specific genome editing is that *Cas9*, expressed in a tissue-specific manner, can cause mutations in cells at the earliest stages of tissue or organ development. For instance, the Arabidopsis *GATA23* promoter (pAtGATA23) [94] is active exclusively at the earliest stages of lateral root initiation (LRI) [91,95], and genes responsible for LRI in Arabidopsis and other higher plants can be edited at the earliest stages of LRI using *Cas9* expression under the control of pAtGATA23 [91]. We have recently identified several genes expressed during the early stages of LRI in plants such as squash (*Cucurbita pepo*) and cucumber (*Cucumis sativus*) that display an alternative root branching mechanism [96], namely *GATA24* and *MEMBRANE-ASSOCIATED KINASE REGULATOR4* from squash (*CpGATA24* and *CpMAKR4*) [97] and *RAPID ALKALINIZATION FACTOR34* from cucumber (*CsRALF34)*. Studies of the expression of pCpGATA24, pCpMAKR4 [97], and pCsRALF34 (Figure 3) promoter reporter fusions in the parental root tip have shown that, in a similar way to pAtGATA23, these promoters are active in the earliest stages of LRI, and therefore can be used for tissue-specific *Cas9* expression in LRI studies involving Cucurbitaceae species.

Translational enhancers can be used to increase Cas9 translational efficiency, such as the enhancers from the Cowpea Mosaic Virus 5′- and 3′-untranslated regions (UTRs) employed for CRISPR/Cas9 genome editing in hairy roots of Abyssinian mustard (*Brassica carinata*). The CaMV 5′-UTR (omega enhancer) was applied in belladonna, the legume *L. japonicus*, potato (*Solanum tuberosum*), and tomato. The 5′-UTR of the Arabidopsis *alcohol dehydrogenase* (*AtADH*) gene (TAIR ID: AT1G77120) was used as a translational enhancer for genome editing in soybean hairy roots (for enhancers, see Table S1 [4,5,6,24,53,76]).

**Figure 4:**
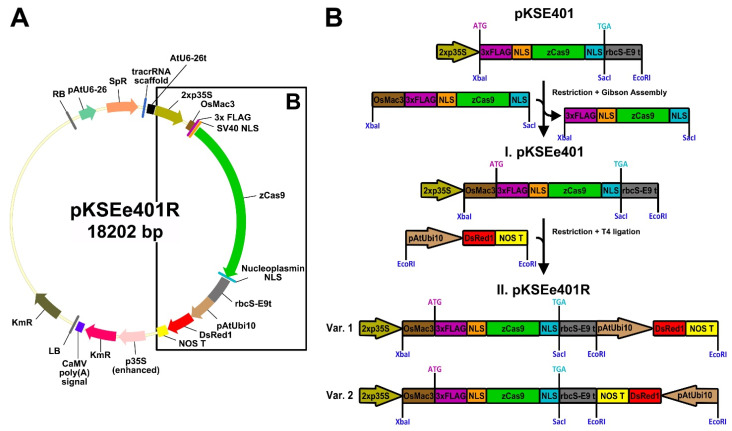
**pKSEe401R plasmid.** (**A**) Map of pKSEe401R; parts (clockwise): RB—right T-DNA border; pAtU6-26—promoter of the Arabidopsis small nucleolar RNA (*snoRNA*) *U6-26* gene; SpR—gene encoding spectinomycin resistance; tracrRNA scaffold—*trans*-activating *crisprRNA* (conservative, used as a binding scaffold for Cas9); AtU6-26t—terminator of the Arabidopsis *snoRNA U6-26* gene; p35S—35S promoter from the Cauliflower Mosaic Virus (CaMV); OsMac3—158 bp fragment of the *OsMac3* 5′-UTR; 3xFLAG—sequence encoding protein tag with the amino acid sequence DYKDHDGDYKDHDIDYKDDDDK; NLS—nuclear localization signal; SV40 NLS—NLS derived from Simian Virus 40 T antigen; zCas9—maize codon-optimized CRISPR associated nuclease 9; rbcS-E9 t—terminator from *pea ribulose-1,5-bisphosphate carboxylase small subunit (rbcS) E9* gene; pAtUbi10—promoter from the Arabidopsis *polyubiquitin 10* gene; DsRed1—gene encoding orange fluorescent protein DsRed1 from *Discosoma* sp.; NOS T—nopaline synthase terminator; KmR—gene encoding kanamycin resistance; CaMV poly(A) signal—polyadenylation signal from CaMV (often used as terminator sequence); LB—left T-DNA border. pKSEe401R nomenclature: p—plasmid; K—KmR; S—p35S; e4—zCas9 enhanced with *OsMac3* 5′ UTR; 01—pAtU6-26p; R—DsRed1. (**B**) pKSEe401R was constructed in two steps: (**I**) cloning of the 158 bp *OsMac3* 5′-UTR fragment into pKSE401 using Gibson Assembly resulted in pKSEe401; (**II**) insertion of the DsRed1 cassette into pKSEe401 using T4 ligation resulted in pKSEe401R with the DsRed1 cassette in either in tandem (**Var. 1**) or in reverse (**Var. 2**) orientation relative to the Cas9 cassette.

Another translational enhancer is the 5′-UTR of the *Mac3* gene from japonica rice (*OsMac3*) [98]. An *OsMac3* 5′-UTR fragment (from −158 to −1 bp before the ATG) [99] was shown to exhibit sufficient activity as a translational enhancer [98] and can be used to improve genome editing in both monocots [99] and dicots [100]. The *OsMac3* enhancer has been used in stable transformants. To enhance the translational efficiency of Cas9-based genome editing in hairy roots, we modified pKSE401 [101] to include the *OsMac3* 5′-UTR fragment (Figure 4A,B). The *OsMac3* 5′-UTR sequence was cloned from genomic DNA of japonica rice *O. sativa* cv. Flagman (Rice Research Institute, Krasnodar, Russian Federation), and then fused with the maize codon-optimized *Cas9* (*zCas9*) from pKSE401 [101] via Gibson Assembly (Gibson Assembly^®^ Master Mix, New England Biolabs, Ipswich, Massachusetts, USA) [102]. The resulting plasmid was named pKSEe401 (Figure 4B), with the “e4” in pKSEe401 representing enhanced *zCas9*, in contrast to the unenhanced *zCas9* previously indicated by the digit 4 in pKSE401 [101].

The second important component of a CRISPR/Cas cassette is the *Cas* gene. As a rule, the Cas9 endonuclease of *Streptococcus pyogenes* and its modified versions are used for targeted gene editing; however, other Cas nucleases (e.g., SpRY or Cas12 versions) have also been employed [103,104,105]. For effective editing in plants, a *Cas* gene should be codon-optimized for use in dicots/monocots, or at least for the plant kingdom. However codon optimization of a *Cas* gene is not strictly required, because it does not guarantee 100% efficiency of genome editing. Moreover, there are examples where codon optimization was not performed, but editing was effective nonetheless. In 12 of the 78 studies examined, zCas9 was used for genome editing in dicots, including Madagaskar periwinkle (*Catharanthus roseus*), soybean, liquorice (*Glycyrrhiza glabra*), Chinese liquorice (*G. uralensis*), and the legume *Medicago truncatula* (Table S1). Furthermore, in 19 of the 78 evaluated studies, a *Cas9* gene codon-optimized for humans was successfully used for hairy root-based genome editing (Table S1). The efficiency of genome editing can, however, be increased by introducing introns in the *Cas9* gene [106]. In addition, nuclear localization signals (NLSs) (two are sufficient) must be attached to the *Cas9* open reading frame (ORF) to ensure its nuclear targeting [107] (Figure 2).

Although there are several CRISPR/Cas technologies based on different Cas activities [104,108], the underlying molecular mechanism is the same in all cases. The two main components of such systems are the gRNA and the Cas nuclease. The gRNA consists of the following two parts: the *crisprRNA* (crRNA) and the *trans*-activating crRNA (tracrRNA). The gRNA binds to the target genomic DNA in front of a specific protospacer adjacent motif (PAM). The fate of the target genomic DNA sequence linked with the gRNA/Cas ribonucleoprotein complex depends on the type of Cas activity.

#### 3.1.1. Genome Editing Based on Double-Strand Breaks in DNA Caused by Cas9 Activity

**Figure 5:**
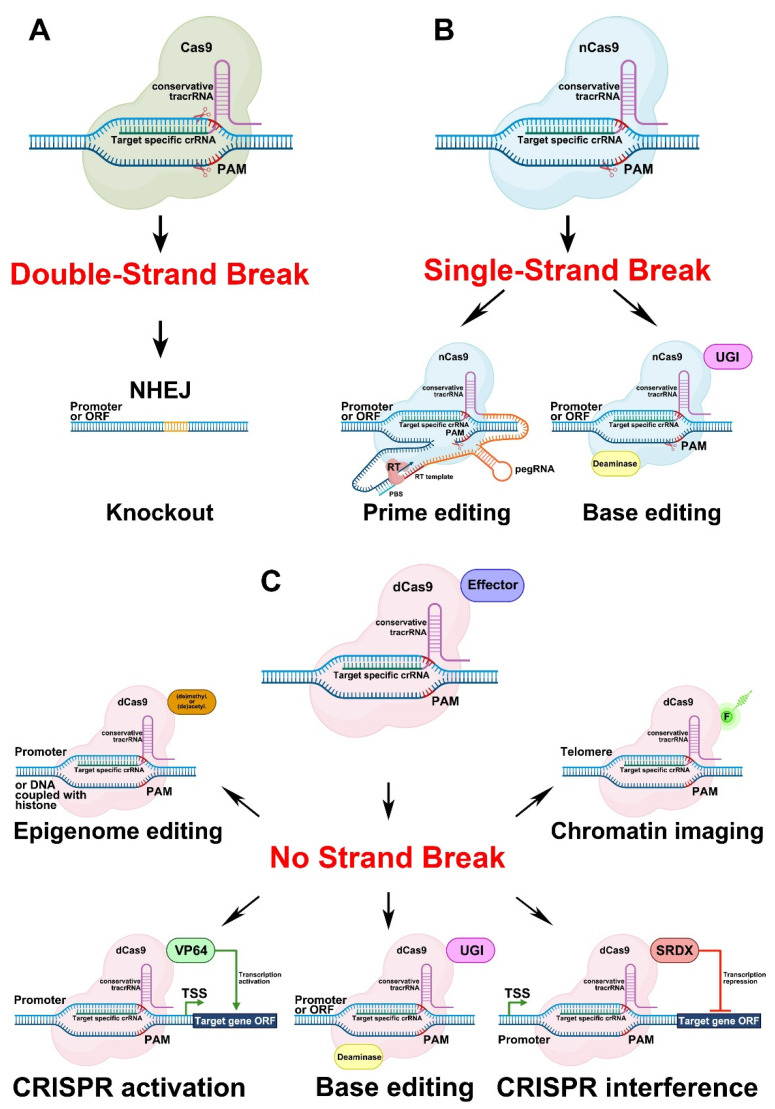
**Types of the CRISPR/Cas9 systems.** (**A**) System based on the Cas9 form producing a double-strand break, NHEJ—non-homologous end joining. (**B**) Approaches using nickase Cas9 (nCas9) activity resulting in a single-strand break, pegRNA—prime editing guide RNA comprised of a target specific crRNA, a conservative tracrRNA, a PBS—primer binding site, an RT template—RNA template for reverse transcription (direction of reverse transcription indicated by a dark blue arrow). The linker joining crRNA and tracrRNA with PBS and the RT template is indicated in orange. RT—reverse transcriptase; UGI—uracil-DNA glycosylase inhibitor domain. (**C**) Techniques using the catalytically inactive (dead) form of Cas9 (dCas9); types of effectors used in dCas9-based systems: (de)methyl.—methyltransferase or demethylase for epigenetic modifications of DNA; (de)acetyl.—acetyltransferase or deacetylase for epigenetic modifications of histones; F—fluorescent protein; VP64—tetrameric repeat of the minimal activation domain of herpes simplex viral protein 16; UGI—uracil-DNA glycosylase inhibitor domain; deaminase—cytidine or adenosine deaminase; SRDX—SUPERMAN Repression Domain X. For systems based on Cas9 and nCas9 activities, scissors indicated in red show the place where DNA strand breaks occur. Figures were created with BioRender.

The most frequently used genome editing system involving Cas is based on the generation of double-strand breaks (DSBs) in the target DNA (Figure 5A). After the introduction of DSBs, non-homologous end joining (NHEJ), a DNA repair mechanism [109], leads to the development of different insertions or deletions (indels) in double-stranded DNA, thereby resulting in lesions in the ORFs (frameshifts) or promoter regulatory elements of target genes.

Genome editing via the introduction of DSBs in the ORFs is the most commonly employed of the CRISPR/Cas-based methods in hairy roots (it was applied in 76 of the 78 studies examined; Table S1). In contrast, two of the 78 reports examined do not concern changes in ORFs. One of these entailed large chromosomal deletions without ORF changes in soybean hairy roots (see Table S1 [37]), while the other involved the editing of a promoter region in tomato hairy roots (see Table S1 [77]). Here, a G-box element targeted by the transcription factors (TFs) MYC1/MYC2/GAME9 in the promoter of the tomato cholesterol biosynthesis gene *STEROL C-5(6) DESATURASE 2* (*C5-SD2*) was successfully edited.

#### 3.1.2. Genome Editing Based on Single-Strand Breaks in DNA Caused by Cas9 Activity

Another group of CRISPR/Cas approaches uses the nickase form of Cas9 (nCas9), which causes single-stranded breaks (SSBs) in DNA. This group comprises two types of genome editing systems—prime editing (PE) and base editing (Figure 5B).

The PE approach was introduced in 2019 for mammalian cells [110] and has been widely used in different fields of biology since its invention. The PE complex consists of two components, namely, a Cas nickase (nCas9) fused with a modified reverse transcriptase (RT) via a linker (Figure 5B). The PE gRNA (pegRNA), in turn, also comprises several parts fused into a single sequence—the crRNA, the tracrRNA, the linker sequence, the primer binding site (PBS), and the template for reverse transcription (Figure 5B). Directed by the pegRNA, nCas9 creates an SSB in front of a PAM in the target genomic DNA sequence. The PBS, which is complementary to the DNA strand, binds to the target sequence in front of the SSB. The nucleotide sequence behind the PBS is used as a template by the RT (Figure 5B). The new sequence formed by the RT can be reincorporated into the target DNA, which leads to mutations, or can be removed by exonuclease activity, which means that the target sequence remains unchanged [110,111,112]. Cases of PE have been reviewed for both transient (protoplasts) and stable plant transformation systems; however, the efficiency of this technology in plants was found to be relatively low [103].

Two classes of enzymes fused with nCas9 are applied for base editing, cytidine (CD) and adenine (AD) deaminases. Such fusions result in the development of the two types of base editors, cytosine (CBE) [113] and adenine (ABE) base editors [114] (Figure 5B). The CBE can change cytosine (C) to thymine (T) or guanine (G) to adenine (A) in the complementary DNA strand, while the ABE performs the opposite substitutions, i.e., T to C or A to G. Briefly, the base editing mechanism works as follows: nCas9, fused with an appropriate deaminase, is directed by the gRNA to the target DNA site and creates an SSB. Deaminase transforms the appropriate nucleotide on the DNA strand with the SSB. The CD transforms C to uracil (U) and U, in turn, is converted to T via DNA repair or replication [113]. To prevent the reversion of U to C via base excision repair, the uracil-DNA glycosylase inhibitor (UGI) domain is included in the CBE (Figure 5B). AD converts A to inosine (I), which is recognized as G by polymerases [114]. The edited strand with the changed nucleotide is then used as a template by the repair mechanism. The development of the base editing technology resulted in improvements in the CBEs [115,116,117,118,119] and ABEs [120,121] characteristics, such as reduced off-target activity, indel frequency, and nCas9 expression levels. Recently, double base editors with CD and AD activities were also developed, first for mammalian cells [122,123,124,125], and then for plants [126,127].

The applications of prime or base editing approaches have been reviewed for some plant species [103,128,129]. Prime and base editing are more accurate but less efficient than the repair of DSBs resulting from Cas9 activity. These approaches need to be optimized to enhance efficiency when used in plants [103,129], and may explain why no studies using prime or base editing on hairy roots have been published to date. Nevertheless, cases of prime or base editing have been reported for several species, including tomato, cotton (*Gossypium hirsutum*), *Nicotiana benthamiana*, potato, rapeseed (*Brassica napus*), and watermelon [103,128,129], for which hairy root transformation protocols are now available [8,130,131,132,133,134]. Besides prime or base editing, nCas9 can be used for gene knock-out, similar to how it was described for potato hairy roots (see Table S1 [79]).

#### 3.1.3. Genome Editing Based on a Version of Cas9 That Causes no Strand Breaks

The catalytically inactive (dead) form of Cas9 (dCas9) has also become an important instrument for biologists. The effect of the dCas9-based system depends on the effector fused to the dCas9. Among effectors, there are activation (AD) or repressor (RD) domains, base editors, epigenome modificators as well as fluorescent proteins [135] (Figure 5C). 

Similarly to nCas9, dCas9 can be fused with deaminases (Figure 5C), but the base editing efficiency of dCas9–deaminase systems is lower than that of systems involving nCas9 [113]. Nevertheless, base editing using dCas9 has also been reported for plants [136,137]. 

Another application of dCas9 is the creation of CRISPR activator (CRISPRa). The principle underlying the activity of CRISPRa is based on DNA binding by an AD fused to dCas9 and directed to the target sequence by the gRNA, which leads to the activation of the expression of the target gene (Figure 5C). The most commonly used AD for fusion with dCas9 is derived from the herpes simplex viral protein 16 (VP16) (UniProt ID—P06492) [138]. The AD represents a tetrameric repeat of the minimal VP16 AD (DALDDFDLDML) separated by glycine–serine linkers (GS), termed VP64 ([DALDDFDLDML]-GS-[DALDDFDLDML]-GS-[DALDDFDLDML]-GS-[DALDDFDLDML]) [139].

The CRISPR interference technology (CRISPRi) is based on the principle, that gRNA/dCas9 ribonucleoprotein (RNP) complex interfere with RNA Polymerase II following block of transcription initiation or elongation. The dCas without any repressor domain (RD) was effective for bacteria, but not for yeast and mammalian cells [140]. Apparently, it is less effective for plants too; therefore, to improve repression efficiency of CRISPRi in plants the additional RD started to use. The RD originates from investigations of the repressor activity of a 12-aa sequence (LDLDLELRLGFA) from the Arabidopsis SUPERMAN protein (TAIR ID: AT3G23130) [141]. The fusion of these 12 aa from SUPERMAN (SUPERMAN Repression Domain X [SRDX]) to the C-terminus of other TFs turned them into strong repressors [142]. 

The CRISPRa and the CRISPRi systems, based on fusions of VP64 or SRDX with dCas9, respectively (Figure 5C), were adapted for plants in 2015 [143,144]; however, compared with the use of CRISPR knockout systems, activator or repressor systems are still rarely used in plant science [145,146,147,148]. Besides VP64 and SRDX, several other ADs [149] and RDs [150] are still awaiting co-application with CRISPR technology or were only rarely used, such as EDLL [143,147], TAL AD (TAD) [143], and modified ERF2 (ERF2m) [145].

dCas9 can also be used for epigenome editing as well as for chromosome visualization (Figure 5C). In the former, dCas9 can be directly fused to different DNA methyltransferases/demethylases or histone acetyltransferases/deacetylases (Figure 5C) [151]. Several studies on epigenome editing in plants have been reviewed previously [151]. Direct fusions of dCas9 with fluorescent proteins have been used for chromosome imaging (Figure 5C) [152,153,154].

In addition, the dCas9-based systems can be classified according to the strategies used to recruit effectors for improving the efficiency of dCas9-based system. The greatest variety of approaches was reviewed for CRISPRa and CRISPRi [135,140]. Reviewed strategies can be extrapolated to other effectors (e.g., base editors, epigenome modificators as well as fluorescent proteins). Multiple copies of the same effector, or different effectors linked in tandem can be used. Another approach is the so-called synergistic activation mediator (SAM) system, based on the co-expression of the dCas9 with modified gRNA and different effectors fused with special proteins that recruit effectors to the dCas9/gRNA RNP complex through their RNA-binding activity [155]. The SunTag system is based on the co-expression of epitope-tagged dCas9 and effectors fused to the corresponding antibody [156]. The effector is attracted to the dCas9/gRNA RNP complex trough the recognition of epitope by the antibody.

Despite the successful use of some effectors in hairy roots, e.g., of SRDX [157,158] or fluorescent proteins [97,159] outside the dCas9-based system, we note that neither these techniques, nor base and prime editing, has yet been commonly applied for hairy roots. Nevertheless, we found the one attempt to obtain carrot hairy roots harboring the SAM system for telomere imaging [154], but results about SAM efficiency in hairy roots were not reported, rendering it difficult to evaluate the efficiency of SAM system for telomere imaging in hairy roots.

### 3.2. gRNA: Design and Testing

The cassette expressing the gRNA in the CRISPR/Cas vector comprises the promoter of the small nucleolar RNA (*snoRNA*) gene, the gRNA sequence, and the *snoRNA* transcription terminator (Figure 2). The gRNA sequence, in turn, consists of two fused sequences, namely, the 17–20-bp *crisprRNA* (crRNA) and the 80-bp *trans*-activating crRNA (tracrRNA) (Figure 2). The crRNA is complementary to the target genomic DNA; thus, its sequence is variable and study-specific. The sequence of the tracrRNA is conserved and functions as a binding scaffold for a Cas nuclease; consequently, it is usually already incorporated in the CRISPR/Cas vector backbone.

*snoRNA* gene promoters are recognized by both RNA polymerase II (U-*snoRNA* classes U1, U2, U4, and U5) and RNA polymerase III (Pol III) (U-*snoRNA* classes U3 and U6) [160,161]. Given that transcribed gRNAs should remain in the nucleus to guide Cas9 to the target genomic DNA sequence, only *snoRNA* gene promoters recognized by Pol III are used in CRISPR/Cas systems. Different Pol III promoters are normally used for expression of several gRNAs in one CRISPR cassette (Figure 2). When assembling multiple gRNAs, the strategy of using different Pol III promoters greatly increases the size of the cloned CRISPR cassette. Meanwhile, vectors using a polycistronic gRNA strategy based on different processing systems have also been published. The expression of multiple gRNAs in a polycistronic cassette is driven using only one Pol III promoter. A single gRNA can be formed and later cleaved either by systems based on tRNA processing mechanisms [162] or by RNA endoribonuclease Csy4 from *Pseudomonas aeruginosa* (Csy4) [163]. The tRNA processing system, which exists in all living organisms, precisely cleaves at the end of a tRNA precursor sequence added to gRNA. Unlike the tRNA processing mechanism, the Csy4 activity does not occur naturally in plants. Therefore, its ORF must be fused to the *Cas* ORF via an ORF encoding a short, self-excising peptide, 2A. Despite the advantages of a polycistronic CRISPR cassette, such as the reduction of the size of constructed vector and an increase in the number of gRNAs, this strategy of genome editing has rarely been used on hairy roots (5 of the 78 studies examined), compared to the use of a CRISPR cassette with different Pol III promoters for expression of several gRNAs (for tRNA and Csy4 processing mechanisms see Table S1 [4,5,53,76,79]).

Active promoters of plant *snoRNA* genes were first characterized in Arabidopsis in the 1990s. Five *U6* genes and promoters were identified—*U6-1* (TAIR ID: AT3G14735), *U6-26* (TAIR ID: AT3G13855), *U6-29* (TAIR ID: AT5G46315) [160], *U6-22* (GeneBank ID LR782545.1; position: plus strand, 13922611–13922735 bp), and *U6-25* (GeneBank ID LR699773.1; position: minus strand, 16444343–16444219 bp) [161]; and three *U3* genes, namely, *U3a* (GeneBank ID LR782546.1; position: minus strand, 22165827 to 22165610 bp), *U3b* (TAIR ID: AT5G53902), and *U3c* (GeneBank ID LR699773.1; position: plus strand, 484549 to 484765 bp) [161]. Studies describing *snoRNA* genes and their promoters in monocots, including wheat (*Triticum aestivum*) (*TaU6* and *TaU3*) [164] and rice (*OsU3*) [165], appeared later.

An avalanche-like increase in plant genome sequencing data [166], together with the first reports on genome editing in several plants species, including Arabidopsis, *N. benthamiana,* rice, sorghum (*Sorghum bicolor*), and wheat in 2013 [7], piqued the interest of researchers in the search for species- or family-specific promoters of *snoRNA* genes to improve genome editing in different plant species. Promoter sequences of *snoRNA* U6 and U3 genes (from here on abbreviated as pU6 and pU3, respectively) were identified in the genomes of a broad range of plant species, followed by the testing of their applicability for genome editing in the corresponding species. Four pU6 sequences were identified in the cucumber genome [167]; three pU6s were identified in cotton [168]; two pU3s and two pU6s were identified in grapevine (*Vitis vinifera*) [169]; six pU6s and one pU3 were identified in maize [170]; one pU6 was identified in stiff brome (*Brachypodium distachyon*) [171]; one pU6 was identified in Douglas fir (*Pseudotsuga menziesii*), which was used for CRISPR/Cas9 editing in radiate pine (*Pinus radiata*) [172]; one pU6 was identified in liverwort (*Marchantia polymorpha*) [173]; and one pU6 was identified in spreading earth moss (*Physcomitrium patens*) [174], in addition to others. Despite the wide variety of pU6/pU3 sequences described, changing from a widely used pU6/pU3 (e.g., pAtU6, pMtU6, pGmU6, or pOsU3) to a species-specific pU6/pU3 version does not guarantee improved genome editing efficiency, similar to what has been observed regarding the uncertain effects of Cas codon optimization. Nevertheless, pU6/pU3 sequences should be at least class-specific (for monocots or dicots) or division-specific (for Bryophyta, Coniferophyta, or Marchantiophyta).

Recognition of the target DNA by Cas9 is directed by the variable part of the gRNA, the crRNA, which is study-specific. In the simplest approximation, the search for protospacers that can potentially be transcribed into crRNA, proceeds as follows: first, an appropriate PAM should be identified. The canonical PAM comprises the sequence 5′-NGG-3′ in either the sense or antisense DNA strand. An oligonucleotide sequence consisting of 17–20 bases adjacent to the 5′-end of the PAM serves as the protospacer, and it can be included in the CRISPR/Cas vector by fusion without any spacers in front of the 5′-end of the tracrRNA (Figure 2).

Although the 5′-NGG-3′ PAM is widely utilized, it is not the only PAM used for genome editing, but merely the target of a subset of CRISPR/Cas systems. Cas variants that recognize other PAMs exist. Different Cas types have been previously reviewed, such as the SpRY variant that recognizes 5′-NGD-3′ or 5′-NAN-3′ (D: A, G, or T); the XNG-Cas9 variant that specifically recognizes 5′-NG-3′ or 5′-NAN-3′; and the iSpyMacCas9 variant that detects the A-rich PAM 5′-NAA-3′ [103,104,105]. Several Cas nucleases that recognize non-canonical PAMs have been used for genome editing in hairy root systems (see Table S1 [37,77]). The Cpf1 (also known as Cas12) nuclease of *Lachnospiraceae* bacterium (LbCpf1) and its temperature-tolerant variant (ttLbCpf1), which displays extended target recognition in T-rich PAM sequences (such as 5′-TTTN-3′), were used both for precise ORF editing and the induction of large chromosomal deletions in soybean hairy roots (see Table S1 [37]). Cas9(VQR), recognizing the 5′-NGA-3′ PAM, was successfully utilized for genome editing in the promoter region of the tomato cholesterol biosynthesis gene *C5-SD2* (see Table S1 [77]).

The knowledge accumulated on plant genome editing has indicated that, besides an appropriate PAM, several other factors are also important in gRNA design (reviewed in [175,176]). Nucleotides complementary to the crRNA sequence should be present in both DNA strands. The size of the crRNA can vary from 18 to 24 bp (19–20 bp are common). The crRNA sequence should not contain poly(T), which represents the transcription termination signal for RNA polymerase III. At least two crRNAs per target gene should be designed in case one is unsuitable. The crRNA should have a high GC content (50–70%) to increase crRNA-target DNA hybrid stability. The crRNA sequence should not pair with more than six nucleotides of the tracrRNA scaffold. The use of a specific first nucleotide (A for pU3 and G for pU6) after *snoRNA* gene promoters enhances the expression and stability of the gRNA. If multiple gRNAs are arranged in a single expression vector, the loss and rearrangement of gRNA components can be avoided by using different RNA polymerase III-dependent promoters. Target specificity is determined by the first 10 nucleotides upstream of the PAM in the crRNA sequence [175,176].

The design and testing of gRNAs can be performed both *in silico* and *in planta*. The rapid growth of plant genome editing-related data, combined with the development of bioinformatics algorithms, has led to the development of a substantial amount of software that takes into account the factors listed above and helps researchers limit the range of crRNA sequences to be analyzed. Some of these programs have been compared in several reviews [177,178,179]. The WeReview repository for CRISPR bioinformatics tools was created to facilitate the search for and comparison of *in silico* gRNA design tools [180]. Currently, 104 programs are available for this purpose. Because all the programs are combined in a pivot table on the website, the use of table filters allows researchers to choose the appropriate CRISPR tool [180]. We believe that the most convenient resources for assessing the whole variety of factors affecting editing efficiency are CRISPOR [181] and CRISPR-PLANT v2 [182]. These programs are connected with the greatest diversity of sequenced plant genomes and also evaluate most of the above-mentioned factors that influence crRNA efficiency.

Tools for assessing gRNA effectiveness *in planta* are also available. Cas9 nuclease activity and the ability of the crRNA to bind to its target DNA sequence can be evaluated on protoplasts before hairy root [183,184] or stable [185,186] transformation. Another attractive technique for *in planta* gRNA testing is the use of fluorescent protein recovery [187]. Here, the 20–50-bp target DNA sequence chosen for CRISPR/Cas9 genome editing is cloned directly into the ORF of yellow fluorescent protein (YFP), resulting in a frameshift. When leaf epidermis cells are co-bombarded with the resulting YFP vector and the customized CRISPR/Cas9 vector, a DSB is induced at the target site. The imperfect repair of the DSB via NHEJ can restore the YFP ORF, leading to the correct translation of the YFP transcript sequence and, consequently, to YFP fluorescence. To estimate the number of genome editing events that lead to YFP fluorescence recovery, an external control with stable fluorescence, such as an mCherry-expressing vector, can be co-transformed with the YFP and CRISPR/Cas9 vectors. The YFP/mCherry fluorescence ratio is indicative of the Cas9 cleavage activity for the designed gRNA [187]. We believe that the described system could be improved by the assembly of all the components (YFP, mCherry, and CRISPR/Cas9) into a single vector.

Another system based on the green fluorescent protein recovery for detecting genome editing events has recently been developed and tested *in planta* [188]. The main difference from the above-described system is that the biosensors were developed not only for CRISPR knockout system but also for prime and base editing as well as for CRISPRa. However, use of external control with stable fluorescence together with these biosensors was not reported, which may be a disadvantage of new biosensors, since stable fluorescent control helps to assess the efficiency of transformation.

Nevertheless, these systems have a wide range of applications, although one condition has to be strictly met, as follows: the plant species chosen for the test systems and the species for which the gRNA is designed should belong to different plant families, as the native genome target sequence might interfere with the sequence inserted into the vector with biosensor. Similarly, these systems should be used with care if the selected target DNA region(s) is/are conserved across plant families.

Finally, owing to the short time required for hairy root establishment as compared with the regeneration of stably transformed plants, the former system can be used not only for gene function studies but also for the rapid testing of genome editing efficiency. A test of genome editing efficiency using hairy root transformation before stable transformation has been conducted for rapeseed and soybean (see Table S3 [7,15,21,22,25,27,31,32,40,44,46]). Genome editing efficiency was tested using hairy root transformation before the regeneration of whole-genome-edited plants from individual hairy roots in chicory (*Cichorium intybus*), the legume *M. truncatula*, tobacco (*Nicotiana tabacum*), potato, and rubber dandelion (see Table S3 [9,60,62,79,84]).

### 3.3. Markers of Transgenicity Used in CRISPR/Cas Vectors: Old Players, New Tricks

The choice of a marker for a CRISPR/Cas vector is important to allow to distinguish between transgenic hairy roots and wild-type roots. There is a great variety of markers, each with its advantages and disadvantages. Since 1983 when the first transgenic plants were obtained [1], methods for the separation of transgenic from non-transgenic material have become well established. Markers for transgenicity of transformed plants can be divided into two large groups, namely, selectable and (visually) screenable markers [9]. The use of selectable markers is based on the principle that plants not carrying the transgene insert will be eliminated following the administration of antibiotics, herbicides, or other phytotoxic compounds, whereas, screenable markers allow the separation of transgenic plants from non-transgenic ones using procedures such as enzymatic reaction-mediated staining of transgenic plant tissues, fluorescence, or pigmentation of transgenic plant parts.

#### 3.3.1. Selectable Markers

Selectable markers were first employed in 1983 (Table S2). The first was the *nptII* gene encoding neomycin phosphotransferase II (NPTII), which provides resistance against two closely related antibiotics, neomycin and kanamycin [189] (Table S2). Subsequent studies on selectable markers led to the identification of enzymes that could inactivate herbicides (Table S2) or nonantibiotic- or nonherbicide-containing phytotoxic compounds [190,191,192] (Table S2), aiming to avoid the introduction of antibiotic resistance genes into crops and, therefore, into the environment. For instance, some markers allow the selection of transgenic plants for their ability to metabolize endogenous carbohydrates (e.g., xylose, mannose) or for their viability on medium containing high concentrations of non-toxic carbohydrates that are normally detrimental to plants [190,191,192] (Table S2). By 2005, at least 17 selectable markers had been identified (Table S2), a number that has since expanded [190,191,192]. In 2021, two new selectable markers were identified—a new algal gene encoding phosphomannose isomerase, which allows regeneration on 0.6% mannose [193], and the *GIBBERELLIC ACID-STIMULATED ARABIDOPSIS 6* (*GASA6*) gene, which allows regeneration on sugar-free medium [194].

Genes encoding enzymes endowing resistance to antibiotics, herbicides, or other phytotoxic compounds are often used as selectable markers for hairy root transformation. Antibiotic resistance genes were utilized in 20 of the 78 studies examined (Table S1), and the selectable *bar* herbicide resistance gene in another 24 studies (Table S1). Thus, enzymes providing resistance to antibiotics or herbicides were used as selectable markers in 44 of the evaluated studies (Table S1). However, while these selectable markers are convenient to use, they share one significant disadvantage. Chimeric roots or organs consisting of transgenic and non-transgenic cells or tissues will not be counterselected because non-transformed cells would be protected from the selective agent by the surrounding transformed cells. Chimeric transgenic hairy roots harboring a CRISPR/Cas T-DNA would consist of a mixture of cells with mutated and non-mutated alleles of the target gene, making it impossible to evaluate the effect of the mutation on target gene function [195].

#### 3.3.2. β-Glucuronidase-Based Screenable Markers

A fundamentally new type of screenable marker, *β*-glucuronidase (GUS) [196] encoded by the *uidA* gene from *Escherichia coli* [197], was discovered in 1986 (Table S2). The GUS enzyme converts a soluble, colorless substrate (5-bromo-4-chloro-3-indolyl-*β*-*D*-glucuronic acid) into an insoluble, colored product (chloro-bromoindigo), which allowed researchers to visually discriminate transgenic from non-transgenic plants [198]. A GUS fusion system, in which GUS constructs also contained a selectable marker, was initially used to study gene expression [196,199,200]. Consequently, in these first studies, GUS could not be considered a screenable marker as transgenicity in transformed plants was detected based on the selectable marker. The first studies using GUS as a screenable marker were performed on maize in 1988 [201], two years after GUS was first used in plants [196] (Table S2). The GUS construct was delivered by particle bombardment to, and detected in, maize cell suspension culture [201]. In 1989, GUS was also used as the screenable marker for the identification of transgenic hairy roots in the legume *Lotus corniculatus* [75].

At this point, nine selectable markers were already known and were routinely used for the identification of transgenic plant material (Table S2); however, several of them could not be used for the transformation of some plant species. For instance, in cabbage (*Brassica oleracea* var. *capitata*), kanamycin and hygromycin were found to inhibit the growth of both transgenic and non-transgenic hairy roots [202]. Kanamycin was also reported to be toxic for not only the formation of transgenic hairy roots, but also for the regeneration of *Rubus* sp. plants from transgenic calli [203]. The antibiotics cefotaxime and carbenicillin similarly prevented the regeneration of *Rubus* sp. plants from calli [203]. Additionally, some plant species chosen for hairy root transformation had natural resistance against antibiotics, as was reported for Brussels sprout (*B. oleracea* var. *gemmifera*) [204]. Therefore, the use of GUS as a screenable marker for hairy root transformation became widespread [75,205,206,207]. Chimeric constructs combining GUS with selectable markers were also generated. Such bifunctional markers combined the enzymatic activity of GUS with the activity of antibiotic- or herbicide-inactivating enzymes. For instance, fusions between GUS and NPTII [208] or between GUS and phosphinothricin *N*-acetyltransferase [209], encoded by the *bar* gene, have been used for the selection of transgenic plants.

Despite the obvious advantages of using screenable markers instead of selectable markers for the detection of genome-edited hairy roots, GUS staining has not been frequently used for this purpose (it was used only in 6 of the 78 studies examined) when compared with antibiotic/herbicide resistance-related selectable markers (Table S1). However, GUS has disadvantages as a screenable marker for the identification of genome-edited hairy roots. Each root must be split into at least three parts, one for GUS staining to confirm transgenicity, one for the isolation of gDNA to confirm genome editing, and one for the evaluation of morphology or metabolite content. Furthermore, if the transgenic root is chimeric, partial staining may lead to wrong conclusions.

#### 3.3.3. Pigment Biosynthesis-Based Screenable Markers

Another subgroup of screenable markers, discovered after GUS, is based on the staining of transgenic material with natural pigments directly synthesized in the transformed tissue in a process that the transgene regulates or participates in. In 1990, the maize *leaf color* gene, encoding a protein that regulates the anthocyanin biosynthesis pathway, was proposed as a screenable marker. The activity of this gene leads to the anthocyanin pigmentation of transformed cells in maize kernels [210] (Table S2). The first studies using anthocyanin pigmentation as a screenable marker were performed on monocots [210,211,212] or used genes cloned from monocots for dicot transformation [213,214]. The potential use of anthocyanin pigmentation in hairy root transformation experiments was evaluated in twelve plant species in 1998 [215]. In short, the use of anthocyanin pigmentation as a screenable marker has been used in plant science since the 1990s.

Further evaluation of anthocyanin pigmentation-based screenable markers showed that genes encoding proteins involved in the regulation of anthocyanin biosynthesis [216], including some of the earliest identified genes from maize [217,218,219] (Table S2), belong to two large gene families, one encoding basic helix–loop–helix (bHLH) TFs and the other myeloblastosis (MYB) TFs [216,220]. Several members of the *MYB* and *bHLH* gene families from different plant species have also been proposed to serve as screenable markers over the last few years. These include the Arabidopsis *PRODUCTION OF ANTHOCYANIN1* (*PAP1*) gene, encoding the MYB75 TF [221]; sweet potato (*Ipomoea batatas*) *MYB1* gene splice variants *a* and *b* [222]; the *MYB10* gene from apple (*Malus domestica* cv. Red Field) [223]; the *bHLH* gene *Delila* from snapdragon (*Antirrhinum majus*) [224]; the *MYBA1* gene from grapevine (*V. vinifera* cv. Merlot) [225]; *Legume Anthocyanin Production 1* (*LAP1*), a member of the *MYB* gene family of the legume *M. truncatula* [226]; the rice *COLORLESS1* gene (*OsC1*), an ortholog of maize *C1* (*ZmC1*) encoding ZmMYB1 [227]; and the *Anthocyanin2* (*AN2*), an rubber tree (*Hevea brassilensis*) ortholog of Arabidopsis *PAP1* as well as of grapevine *MYB1A* [228]. Several of these newly described genes, including *MYB75*/*PAP1* from Arabidopsis [229] and *LAP1* from *M. truncatula* [226,230], have been used as screenable markers for hairy root transformation. Additionally, Arabidopsis *MYB75*/*PAP1* was cloned into a CRISPR/Cas9 vector as a screenable marker for hairy root transformation of soybean (see Table S1 [26]), whereas *OsC1* was used for stable transformation of rice by a CRISPR/Cas9 vector [227]. Preliminary tests on the efficiency of *MYB75*/*PAP1* as a marker showed that purple/red staining in soybean transgenic roots was consistent with GUS staining. Purple-stained soybean hairy roots obtained by CRISPR/Cas9-mediated editing of *Rfg1* were also efficiently detected [229]. It was also reported, that detection of genome-edited rice regenerants by *OsC1* screenable marker was also successful [227]. The diversity of genes regulating anthocyanin biosynthesis [216] leads to a wide choice of potential screenable markers in addition to the six already proposed. Conversely, the diversity of factors involved in the regulation of anthocyanin biosynthesis renders these factors unsuitable as universal screenable markers.

Another candidate for a screenable marker, betalain, was proposed in 2020 [231]. Betalains are red-violet or yellow pigments that accumulate in flowers, fruits, and vegetative tissues of plants in most core families of the order Caryophyllales. If betalains are present in plants, they may replace anthocyanins [232]. Betalain is biosynthesized from tyrosine in a four-step process that involves three genes [231]. The first step is catalyzed by a P450 cytochrome oxygenase, CYP76AD1; the second step is catalyzed by L-DOPA 4,5-dioxygenase (DODA) or, alternatively, CYP76AD1; the third step involves a non-enzymatic, condensation reaction; finally the fourth step is catalyzed by a glucosyltransferase (GT). A construct called RUBY, containing the genes coding for these three proteins combined in a single ORF and separated by the ORF for a short, self-excising peptide, 2A, was proposed as a new screenable marker [231,233]. Arabidopsis and rice plants transformed with the RUBY construct exhibited the red betalain coloration, allowing to distinguish transgenic plants from wild-type plants or calli [231].

Although the idea of an expression system for multiple protein-coding genes based on the use of the 2A self-cleaving peptide seems very attractive, the system does not always function efficiently. Three events are possible during the translation of a transcript containing two ORFs linked by the 2A coding sequence, namely, the independent translation of the two transcripts via a skipping mechanism; the ribosome cannot translate the 2A peptide sequence and is detached from the transcript; and the production of a fusion protein without the 2A peptide [234]. In addition to failures of the 2A self-cleavage system, several other factors can affect the efficiency of the system in plants, including the length of the self-cleaving peptide sequence and the nucleotide composition of the sequences surrounding the 2A sequence. Several scientists believe that the cleavage efficiency should be confirmed by Western blotting [235]. These observations indicate that the use of the RUBY construct can lead to the underestimation of transformation efficiency resulting from failures of the self-cleaving process. We could not find any studies that compared the transformation efficiency of RUBY with that of other constitutively expressed screenable markers (e.g., GUS, fluorescent proteins) in parallel.

Another version of a screenable marker based on the same gene set was proposed in 2021. Here, the transcription of *CYP76AD1*, *DODA*, and *GT* was driven by a constitutive promoter (p35S). *N. benthamiana* leaves infiltrated with a mixture of *Agrobacterium* strains, each of which harbored one of three vectors expressing *CYP76AD1*, *DODA*, or *GT*, exhibited betalain-colored spots. In another study, *CYP76AD1*, *DODA*, and *GT*, each under the control of a tomato fruit-specific promoter (pE8), were cloned into a single vector. The system was effective even though the construct contained three copies of the same promoter. In tomato fruits, dark red betalain coloration could be clearly distinguished from their natural red color [236]. The first use of betalain pigmentation as a screenable marker for hairy roots involved the use of a promoter induced in response to arbuscular mycorrhizal (AM) colonization in the so-called MycoRed reporter system [237]. The MycoRed constructs contained three genes in tandem, i.e., *CYP76AD1* under the control of the legume *M. truncatula* AM-responsive promoters of pMtPT4 (*PHOSPHATE TRANSPORTER4*) or pMtBCP1 (*BLUE COPPER PROTEIN1*), while expression of the *DODA* and *GT* genes was driven by p35S and pUbi10, respectively. The approach allowed in vivo real-time visualization of AM colonization in *M. truncatula* hairy roots because transgenic root parts colonized by *Rhizophagus irregularis* turned purple due to the local induction of betalain biosynthesis.

The use of independent *CYP76AD1*, *DODA*, and *GT* constructs under the control of different promoters [236] allows for better control of their expression levels compared with the version containing a single construct with 2A self-cleavage peptide ORFs [231]. Nevertheless, the use of a construct with three separate cassettes also has disadvantages. The three-cassette combination is long when compared with commonly used marker gene constructs; accordingly, the T-DNA transferred by *Agrobacterium* into the plant genome is also significantly longer, which leads to a reduction in transformation efficiency. It is theoretically possible to generate a construct containing all three betalain synthesis genes and a *Cas9* gene, the expression of which would be driven by bidirectional promoters that drive gene expression in both upstream and downstream directions [238,239]. Two constitutive bidirectional promoters could be used in a CRISPR/Cas9 vector also containing the three betalain biosynthesis genes, allowing the expression of four genes under two promoters. The use of bidirectional promoters in CRISPR/Cas9 systems adapted for plants has been previously reported [240].

To conclude the overview of pigment biosynthesis-based screenable markers, we highlight their advantages and restrictions. As advantages, these markers are non-invasive, which represents a clear advantage over the use of GUS-staining technology. They can be visually screened and allow for the detection of chimerism, an option not available when using selectable markers. However, before these markers can be used, it is necessary to determine whether the hairy roots of a given plant species show natural red or purple pigmentation, such as the shikonin-containing hairy roots of the purple gromwell (*Lithospermum erythrorhizon*) [241,242] and *Arnebia hispidissima* [243]; the purple pigmented hairy roots of Egyptian henbane (*Hyoscyamus muticus*) [244]; the hairy roots of the purple coneflower (*Echinacea purpurea*) when cultured in the light [245]; the betalain-containing hairy roots of red beet (*Beta vulgaris*) [246,247]; the rutin-containing hairy roots of Tartary buckwheat [248]; and the hairy roots of black carrot (*Daucus carota* ssp. *sativus* var. *atrorubens*) when cultured in the light [249].

#### 3.3.4. Fluorescent Protein-Based Screenable Markers

Fluorescent protein (FP)-based screenable markers were the last group of markers introduced for the detection of transgenic plants/tissue (Table S2). The discovery of the GFP from the jellyfish *Aequorea victoria* in 1992 [250] changed the world of screenable markers. However, the native *gfp* gene exhibited low levels of expression and, in plant cells, the encoded protein displayed low fluorescence. To improve *gfp*, modified versions of the gene were used, such as synthetic *gfp* (*sgfp*) [251], modified *gfp* (*mgfp4*) [252], and soluble-modified *gfp* (*smgfp*) [253]. The first report on the use of GFP as a screenable marker without the application of any additional already known markers (e.g., antibiotic- or herbicide resistance gene, or *GUS*) occurred in April 1995 [254] (Table S2). Protoplasts of sweet orange (*Citrus sinensis*) were transformed either with p35S-GFP alone or together with a p35S-GUS construct, with green fluorescence being detected in both cases [254]. The screening of whole Arabidopsis plants by GFP fluorescence and in the absence of a selectable marker was reported two years later [252], while the successful use of GFP for the detection of transgenic hairy roots of the legume *L. japonicus* was reported only in 2003 [255]. The range of FP-based screenable markers was expanded when a new red FP, DsRed1, was obtained from *Discosoma* sp. in 1999 [256] (Table S2). DsRed1 was first used as a screenable marker for the identification of transgenic tobacco protoplasts [257] (Table S2), but its application as a screenable marker for transgenic plants was gradually expanded, such as for the detection of transgenic Arabidopsis seeds [258]. The use of DsRed1 as a screenable marker in hairy root transformation experiments was demonstrated for the legumes *M. truncatula* [159] and soybean [259], as well as for *Datisca glomerata*, a plant with roots with a thick periderm [260].

FPs are often used as components of bifunctional markers. To expand the applicability of selectable markers, GFP was fused with several proteins, including aminoglycoside-(3″)(9)-adenylyltransferase [261], NPTII [262], blasticidin *S deaminase* [263], and phosphomannose isomerase [264]. Furthermore, GFP–GUS fusions are frequently used as screenable markers [265].

FP-based screenable markers seem ideal in the context of CRISPR/Cas9 genome editing combined with hairy root transformation. First, they can be used to distinguish chimeric from fully transgenic roots in a non-invasive manner. Second, FP-based fluorescence can be stronger than any intense natural pigmentation of obtained hairy roots, allowing even transgenic hairy roots with natural pigmentation to be easily identified. Finally, FP-based screenable markers have wide applicability in combination with CRISPR/Cas9 vectors. They not only allow the identification of transgenic hairy roots, which can have positive genome editing events, but can also be used to identify transgenic seeds [167,266,267,268,269,270,271] obtained from regenerants of the same hairy roots. The Cas9 nuclease-encoding gene must be removed from the genome of plants regenerated from explants to prevent non-specific genome editing in future generations. Accordingly, seeds obtained from regenerated plants will be selected for the absence of the screenable marker, i.e., only seeds that do not show fluorescence and, therefore, do not have a T-DNA insert with an active *Cas* gene will be selected.

Notably, the number of available FPs has continued to grow, and the palette is not limited to GFP and DsRed1. Researchers can choose from an appropriate spectrum of FP-based screenable markers using the FP database (FPbase) [272]. Overall, it is difficult to find disadvantages associated with FP-based screenable markers. The one drawback might be that expensive equipment such as a fluorescence stereomicroscope with an appropriate set of filters is required for the detection of fluorescence, which may explain why FP-based screenable markers were only used for genome editing using hairy roots in 23 of the 78 studies examined (Table S1).

#### 3.3.5. New Strategies for the Insertion of a Marker Cassette into a CRISPR/Cas Vector

In addition to the type of marker used, strategies for the insertion of a marker cassette into the CRISPR/Cas vector backbone should also be considered in vector design. Two strategies are currently possible. The first one involves the insertion of a separate cassette consisting of a constitutive promoter, a marker gene, and a transcription terminator into the construct. This strategy has been successfully applied in most CRISPR/Cas9 studies using hairy root transformation (74 of the 78 studies examined) (Table S1). The second strategy involves the fusion of the *Cas9* gene with the gene encoding the marker (selectable or screenable). Depending on the sequence between two ORFs, a Cas9 protein can be produced linked to marker protein via a linker, or both proteins can mature independently.

The fusion of Cas9 nuclease with a FP through a linker was used in some of the first genome editing studies in plants [7,273]. A GFP fused via a linker to a Cas9 codon-optimized for humans was used to detect transgenic, and thus genome-edited, *N. benthamiana* leaf cells. That this resulted in the successful editing of the *phytoene desaturase* gene [273] suggests that the fusion of GFP with Cas9 did not affect the nuclease activity of the latter. A similar system could be used as a screenable marker for hairy root transformation as the Cas9–FP fusion would be bifunctional, i.e., it could be used both for transgenic root detection and genome editing. However, we did not find any studies using this strategy for hairy roots. The application of this strategy must be precisely tested to ensure that this type of CRISPR/Cas-based system allows the separation of transgenic hairy roots from non-transgenic ones.

The independent maturation of the nuclease and marker protein was proposed by Osakabe et al. first for TALEN [274] and then for Cas9 nuclease [275]. Here, two ORFs are linked by a sequence encoding the self-excising peptide 2A [233]. The resulting chimeric protein is subsequently cleaved into a nuclease (e.g., Cas9) and a marker protein (e.g., FP). Since 2016, the use of this version of the nuclease–marker protein fusion has become widespread for stable plant transformation [91,275,276,277,278,279,280,281,282]. For instance, a Cas9-2A-GFP construct was utilized for genome editing using hairy root transformation (four of the 78 studies examined) in belladonna, potato, the legume *L. japonicus*, and tomato (see Table S1 [4,5,53,76]). Even though successful CRISPR/Cas9 editing of target genes was reported in these studies, the identification of transgenic hairy roots via GFP fluorescence was reported only for *L. japonicus*. For belladonna, transgenic roots were identified based on kanamycin resistance, while for tomato and potato, identification was based on direct PCR-based analysis (see Table S1 [4,5,53,76]). These observations suggest that the efficiency of transgenic hairy root detection through FPs derived from the Cas9-2A-FP chimeric protein varies among plant species, presumably due to the imperfection of the 2A self-cleavage, and requires preliminary analysis using an external control with stable fluorescence.

#### 3.3.6. Construction of the CRISPR/Cas9 Vector with the DsRed1 Screenable Marker

CRISPR/Cas9 vectors carrying FP-based screenable markers are widely used both for the positive selection of transgenic hairy roots (Table S1) and the negative selection of seeds obtained from genome-edited plants [167,266,267,268,269,270,271]. One of the most popular CRISPR/Cas9 vector systems used for genome editing both in hairy roots and in stable transformants is pKSE401 and its relatives (e.g., pBSE401, pHSE40), which allow the simultaneous editing of up to four gene targets [101]. At least 7 of the 78 studies used pKSE401 or pHSE401 modified by the inclusion of several screenable markers. pKSE401 was modified either with GFP [167,267] or GUS [283,284,285], while pHSE401 was modified with GFP [167], AtMYB75/PAP1 (resulting in anthocyanin pigmentation) [229], or GUS [286]. However, no reports exist of pKSE401 or its derivatives being modified to include a red fluorescent protein-encoding gene.

**Figure 6:**
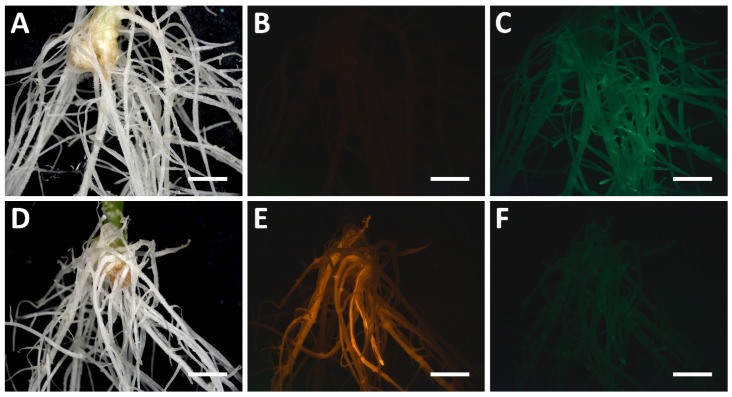
**Cucumber (*Cucumis sativus*) hairy roots co-transformed with pKSEe401R with a DsRed1 cassette in tandem (A–C) or reverse (D–F) orientation relative to the Cas9 cassette.** (**B**) DsRed1 fluorescence was absent when the fluorescent protein (FP) cassette was in tandem orientation relative to the Cas9 cassette; (**E**) DsRed1 fluorescence was visible when the FP cassette was in reverse orientation relative to the Cas9 cassette. (**A**,**D**) bright field illumination; (**C**,**F**) root autofluorescence. Scale bars denote 5 mm.

We modified pKSE401 [101] with the gene encoding DsRed1 (Figure 4B) because its emission spectrum could be clearly separated from the green autofluorescence present in the roots of our model plant *Cucumis sativus* (Figure 6C,F). For this purpose, the pAtUbi10-DsRed1-NOS terminator cassette created by Limpens et al. [159] was amplified from the pK7GWIWGII-RedRoot vector [287,288] with minor modifications. The cassette is 2469 bp long and originally consists of 1523 bp of pAtUbi10 (from −1508 to +15 relative to the ATG of the *AtUbi10* gene); 681 bp of the DsRed1 ORF; and 265 bp of the NOS terminator. We have previously shown that the shorter version of pAtUbi10 (636 bp; from −637 to −1 relative to the ATG of the *AtUbi10* gene) from pCMU-NUCr (AddGene #61168) [289] can also drive the constitutive expression of an FP reporter in transgenic hairy roots [96]. Alignment of the two pAtUbi10 sequences indicated that they were 99.5% identical from position −637 to −1 (relative to the ATG of the *AtUbi10* gene). Accordingly, we designed primers that would amplify approximately half of the AtUbi10 promoter from the original pAtUbi10-DsRed1-NOS cassette (652 instead of 1523 bp) and generated a pAtUbi10-DsRed1-NOS cassette containing the modified AtUbi10 promoter (from −637 to +15 relative to the ATG of the *AtUbi10* gene). The 1598-bp PCR product (pAtUbi10-DsRed1-NOS terminator) was cloned into pKSE401 containing the *zCas9* gene enhanced by the *OsMac3* 5′-UTR (Figure 4B), resulting in a pKSEe401R vector with the DsRed1 cassette in either tandem or reverse orientation relative to the Cas9 cassette (Figure 4B). The DsRed1 expression cassettes (pAtUbi10-DsRed1-NOS terminator) were sequenced and no nucleotide rearrangements or substitutions were observed compared with the original pAtUbi10-DsRed1-NOS sequence.

The resulting vectors were used to transform cucumber (*Cucumis sativus* cv. Kustovoy, Sortsemovoshch, St. Petersburg, Russian Federation) using *A. rhizogenes* strain R1000 as previously described [290]. In total, 54 composite plants with hairy roots were obtained (Figure 6A,D). No DsRed1 fluorescence was observed in 27 composite plants transformed with pKSEe401R containing the DsRed1 cassette in tandem orientation to the Cas9 cassette (Figure 6B). In contrast, DsRed1 fluorescence was detected in each of 27 composite plants with hairy roots harboring the DsRed1 cassette in reverse orientation to the Cas9 cassette (Figure 6E). This indicates that a CRISPR/Cas9 vector with a DsRed1 cassette in reverse orientation can be applied both for the identification of transgenic hairy roots and the selection of seeds from regenerants.

## 4. Possible Applications of CRISPR/Cas9 Genome Editing Using Hairy Root Transformation

**Figure 7:**
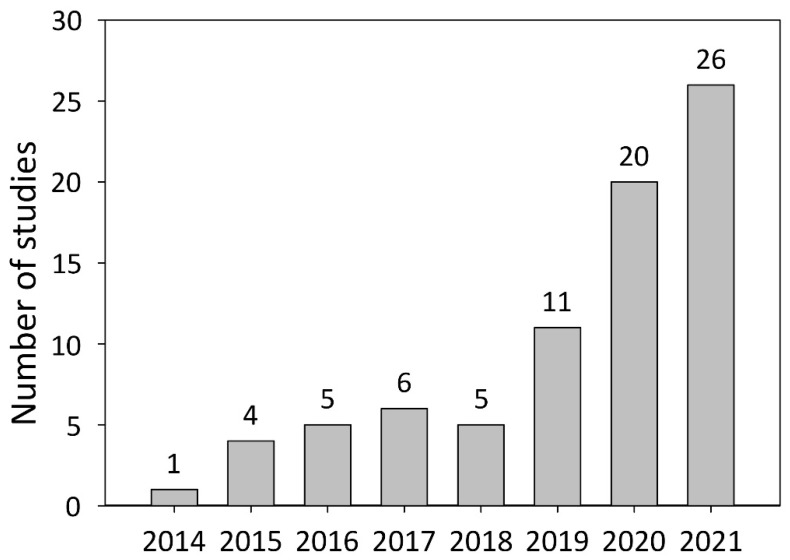
**Number of studies on CRISPR/Cas9 genome editing in hairy roots (analyzed on the 13 December 2021).** Histogram is based on the Google-search of studies presented in Table S1.

An increasing number of studies have reported on the use of CRISPR/Cas9 genome editing since the first application in tomato hairy roots in 2014 [8] (Tables S1 and S3), with four to six studies per year being published between 2015 and 2019 (Figure 7). Altogether, 78 studies combining CRISPR/Cas9 technology and hairy root transformation were undertaken in 26 plant species (Figure 7; Tables S1 and S3).

Two main approaches were used in these studies. The first involved the detection of CRISPR/Cas9 genome editing events in hairy roots without further phenotyping (Table S3). Some of these studies aimed at testing the efficiency of the CRISPR/Cas9 genome editing approach using hairy root transformation for the different plant species. In another group of studies, phenotyping of edited roots was excluded as the main goal was to obtain whole genome-edited plants regenerated from individual edited hairy roots. In other cases, hairy root transformation was used as a system for the rapid testing of crRNA efficiency; gRNAs with maximal efficiency in the screening system were selected for use in stable transformation (Table S3). The second approach involved both genotyping and phenotyping of genome-edited hairy roots (Table S3). Investigations were divided into the following four subgroups according to the aims: root development or root function; root nodule symbiosis; resistance to biotic or abiotic stresses; or metabolic engineering (Table S3). 

## 5. Conclusions and Perspectives

Successful genome editing using hairy root transformation requires several preconditions. First, the genome sequence of the target plant must be known. A large number of sequenced plant genomes [166,291] and plant genomic databases [292,293] are currently available, and the numbers keep growing [294]. Moreover, the absence of a published genome sequence is now less of an obstacle than previously as genome sequencing and assembly have become much cheaper and faster owing to the development of new sequencing technologies and assembly algorithms [295,296].

The second precondition for successful genome editing is a protocol for high-efficiency transformation by *A. rhizogenes* strains and the regeneration of hairy roots. To date, more than 100 plant species have been shown to form hairy roots after infection with *A. rhizogenes* [3,5,10]. While this list is impressive, it means that hairy root transformation protocols have yet to be developed for many plant species. When doing so, attention must be paid to the selection of *A. rhizogenes* strains, some of which can be highly virulent for certain species; the co-cultivation conditions of agrobacteria with a plant; the stage of plant development; and the type of explant. A well-adapted hairy root transformation protocol can increase the transformation and regeneration efficiency of transgenic roots by up to 70% [297]. Another way to improve the efficiency of a hairy root transformation protocol might be the generation of new supervirulent *A. rhizogenes* strains either via introducing additional virulence genes [70] or by genome editing [33].

The third precondition refers to the design of an appropriate CRISPR/Cas vector containing cassettes expressing the *Cas* gene, gRNAs, and transgenicity marker genes. Although different codon-optimized *Cas* genes together with numerous constitutive, inducible, or tissue-specific promoters have been used in plants, the essential components of a CRISPR/Cas vector, in our opinion, are custom-designed crRNA sequences and a cassette with an efficient selectable or screenable marker of transgenicity, as even codon-optimization of a *Cas* gene does not guarantee 100% genome editing efficiency. Additionally, whether the use of species-specific snoRNA *U3*/*U6* gene promoters for gRNA expression affects editing efficiency remains controversial [298,299].

The fourth precondition is the proper design of the gRNA specifically of its variable part, the target-specific crRNA. It remains unknown why some crRNAs have higher mutation rates than others; however, the accumulation of experimental data has led to the development of bioinformatics algorithms that allow the assessment of some of the factors influencing the effectiveness of the chosen crRNA. Thus far, there are 104 programs for *in silico* crRNA design and analysis of its efficiency [180].

The choice of a marker-gene is an important consideration in the design of a CRISPR/Cas vector as the use of an unsuitable marker of transgenicity can lead to the so-called double chimerism problem. The transgenic material might be chimeric both with respect to both the insertion of the artificial T-DNA and the mutations introduced by the CRISPR/Cas system. The first chimerism-related event arises when the marker does not allow to distinguish between completely transgenic roots or calli and chimeric ones that consist of transgenic and non-transgenic tissues. T-DNA chimeras are best avoided by the using screenable markers (e.g., GUS, FPs, anthocyanin or betalain pigmentation). The second event arises because the mutations introduced in the genome by the CRISPR/Cas system are random. Multiple mutations in one or both alleles of the same gene can be introduced in an individual plant or hairy root, and this chimerism can be detected only by sequencing. Examples have been described for both stable [300,301] and hairy root [302,303] transformation.

Not all CRISPR/Cas techniques have been applied in hairy roots. CRISPR/Cas systems with Cas9, Cas9(VQR), LbCpf1, tt-LbCpf1, or nCas9D10A nucleases have only been used to knock out target genes. Other techniques, such as base editing, prime editing, CRISPRa, CRISPRi, epigenome modification, or chromatin imaging, have yet to be successfully combined with hairy root transformation.

## Data Availability

All relevant data are within the paper and its Supplementary Materials files.

## Supplementary Materials

The following are available online at https://www.mdpi.com/article/10.3390/plants11010051/s1: Table S1: List of *Agrobacterium* strains and CRISPR/Cas vector components from studies using hairy root transformation; Table S2: Examples of the old-known conventional and nonconventional markers used for identification of transgenicity in transformed plants; Table S3: List of research tasks solved using CRISPR/Cas editing of different genes in hairy roots.

## Abbreviations

CRISPR	clustered regularly interspaced short palindromic repeats
Cas	CRISPR associated nuclease
crRNA	crisprRNA
tracrRNA	*trans*-activating crisprRNA
PAM	protospacer adjacent motif
ORF	open reading frame
TF	transcription factor
TSS	transcription start site
