# Supplementary material for: Hairy CRISPR: Genome Editing in Plants Using Hairy Root Transformation
Source: Plants (Basel). 2021 Dec 24;11(1):51. doi: 10.3390/plants11010051 (PMC8747350; doi:10.3390/plants11010051)
Supplement: Supplementary file 1 [file plants-11-00051-s001.zip › plants-1505378-supplementary.pdf]

plants

MDPI

Supplementary Materials

Hairy CRISPR: Genome Editing in Plants Using Hairy Root Transformation

Alexey S. Kiryushkin, Elena L. Ilina, Elizaveta D. Guseva, Katharina Pawlowski and Kirill N. Demchenko

Table S1. List of Agrobacterium strains and CRISPR/Cas vector components from studies using hairy root transformation

Plant species	Reference	Year	Agrobacterium strain	Vector name or type of cloning system	Promoters for gRNA expression	Promoters for Cas9 expression	Cas9 type	Gene chosen for genome editing (name or ID)	Marker of transgenic hairy roots
***Arachis hypogaea* (peanut)**	[1]<br>[3]	2019<br>2020	K599	p201B/Cas9 pUC-gRNA [2]<br>p201G/Cas9 pUC-gRNA [2]	pMtU6.6	2xp35S	hCas9	*ahFAD2a*<br>*NFR1A1; A2; B1; B2; NFR5A; B*	Bar<br>EGFP
***Atropa belladonna* (belladonna)**	[4]	2021	ATCC15834	pMgP237-2A-GFP [5]	pAtU6-26	2xp35SΩ	AtCas9 fused with GFP via P2A peptide	*pyrrolidine ketide synthase (PYKS)*	GFP; **<u>Km</u>**[PC]
***Brassica carinata* (Abyssinian mustard)**	[6]	2017	C58 (pRiARqua1)*	pB-CRISPR+35S:: GFP (V112)	pAtU6-26	pPcUbi4-2	hCas9 with translational enhancers from the Cowpea Mosaic Virus 5′ and 3′ UTRs	*FASCICLIN-LIKE ARABINOGALAC TAN PROTEIN 1*	**<u>3xGFP</u>**; PPT
***Brassica napus* (rapeseed)**	[7]	2020	AR15834	pGWB401 based vectors	pAtU6	p35S	BnCas9	*HVA22 homologue C; AGO1*(*miR168*); *TAO1*(*miR1885a*)	Km
***Catharanthus roseus* (Madagascar periwinkle)**	[8]	2021	R1000*[PC]	MoClo system with custom modifications	pAtU6-26	pAtUbi10	izCas9	*Jasmonate-associated MYC2; Jasmonate-associated MYC3; Repressor of MYC2 targets 1*	Hygr
***Cichorium intybus* (chicory)**	[9]	2019	15834	pYLCRISPR/Cas9 P35S-B [10]	pCiU6-1	2xp35S	pCas9	*PDS*	Bar

Plant species	Reference	Year	Agrobacterium strain	Vector name or type of cloning system	Promoters for gRNA expression	Promoters for Cas9 expression	Cas9 type	Gene chosen for genome editing (name or ID)	Marker of transgenic hairy roots
***Eucalyptus grandis* (flodded gum)**	[11]	2020	A4RS	GoldenGate cloning system with custom components	pAtU6	2xp35S	hCas9	*Cinnamoyl-CoA reductase1*; *IAA9A*	DsRed
***Glycine max* (soybean)**	[12]	2015	K599	custom vector	pAtU6	pZmUbi	dpCas9	*bar* gene in *bar* soybean line; *GmFEI1*; *GmFEI2*; *GmSHR*	GFP
	[2]			p201N/Cas9 pUC-gRNA [2]	pMtU6.6	2xp35S	hCas9	*gfp* gene in *gfp* soybean line; *Glyma07g14530*; *01gDDM1*; *11gDDM1*; *miR1509*; *miR1514*; *Glyma04g36150* (*MET1*); *Glyma06g18790* (*MET1*)	Km
	[13]		ARqua1*	pMDC32/GUS/ GmCas9	pAtU6-26	2xp35S	GmCas9	*glutamine synthase 1*; *chalcone-flavanone isomerase 20*	**GUS**; Hygr
	[14]			pCAMBIA3301 based vectors	pAtU6-26; pGmU6-10	p35S	pCas9	*Glyma06g14180*; *Glyma08g02290*; *Glyma12g37050*	Blp
	[15]	2016	K599	Cas9/sgRNA plasmid construction kit (VIEWSOLID Biotechnology)	pAtU6	pZmUbi	dpCas9	*PDS*	Bar
	[16]			custom modified pHSE401 [17]	pAtU6-26	2xp35S	zCas9	*Glyma.01G165800*; *Glyma.01G165800-D*	**GUS**; Hygr
	[18]	2017		pHSE401 [17]	pAtU6-26	2xp35S	zCas9	*Rg1*	Hygr; **visual detection for nodules formation**

Plant species	Reference	Year	Agrobacterium strain	Vector name or type of cloning system	Promoters for gRNA expression	Promoters for Cas9 expression	Cas9 type	Gene chosen for genome editing (name or ID)	Marker of transgenic hairy roots
	[19]	2018		pCAMBIA3301 based vectors	pGmU6	p35S	pCas9	*GmMYB118*	Blp
	[20]			custom modified pKSE401 [17]	pAtU6-26; pAtU6-29	2xp35S	zCas9	*Nod Factor Receptor 1a*	**GFP**; Km
***Glycine max* (soybean)**	[21]	2019	K599	pYLCRISPR/Cas9 P35s-B [10]	pAtU3b; pAtU3d; pAtU6-26; pAtU6-1	2xp35S	pCas9	*LATE ELONGATED HYPOCOTYL 1a; 1b; 2a; 2b*	Bar
	[22]			pFGC5941	pAtU6-26	2xp35S	hCas9	*GmFAD2-1A; GmFAD2-1B*	Bar; **PCR**
	[23]			pZG23C05 (ZGene Biotechnology Inc.)	pAtU6	pUbi	dpCas9	*Glyma.03g163500; Glyma.10g037100; Glyma.10g246300; Glyma.13g123500; Glyma.19g164800; Glyma.19g164900; Glyma.20g146200; Glyma.20g148200; Glyma.20g148400*	Bar
	[24]			pRI201-AN (TaKaRa Bio)	pGmU6	p35S	GmCas9 with AtADH 5′UTR	*Isoflavonoid syntase*	Bar
	[25]	2020		custom modified pCAMBIA1300 based vectors	pGmU6	pM4	Cas9	74 nodulation genes; 27 seed genes	Bar
	[26]			custom modified pHSE401 [17]	pAtU6-26	2xp35S	zCas9	*Rg1*	AtMYB75/PAP1
	[27]			HBT-pcoCas9 [28] pBlu-gRNA [13]	pAtU6-26	2xp35S	pCas9	*galactinol synthase* (*GOLS*) genes: *GOLS1A*; *GOLS1B*	Bar
	[29]			pHairyRed [30]	pGmU6	p35S	pCas9	*GmPHR1; GmPHR4*	Hygr; **DsRed2**
	[31]			Tri-KO1, Tri-KO2, Tri-KO3	pGmU3; pGmU6	pGmUbi3	dpCas9	*GmF3H1; GmF3H2; GmFNSII-1*	Bar

Plant species	Reference	Year	Agrobacterium strain	Vector name or type of cloning system	Promoters for gRNA expression	Promoters for Cas9 expression	Cas9 type	Gene chosen for genome editing (name or ID)	Marker of transgenic hairy roots
	[32]			custom modified pCAMBIA3300 based vectors	pAtU6-26; pAtU3b1; pAtU6-1; pAtU3d; pAtU6-29; pAtU3b2	2xp35S	Cas9	*GmAGO7a*; *b*	**Bar**; **EGFP**
***Glycine max* (soybean)**	[33]	2021	ARqua1*	p201G/Cas9; pUC-gRNA [2]	pMtU6.6	2xp35S	hCas9	*Glyma15G191200*	EGFP
	[34]		K599	p201-EGFP-C9	pGmUbi3; pMtU6	pGmUbi3	hCas9	*GmIPK1*; *GmIPK2*	EGFP
	[35]			pFGC5941	pU6	p35S	Cas9	*Glyma06g14180*	Bar; **GUS/GFP**
	[36]			p201G/Cas9; pUC-gRNA [2]	pMtU6.6	2xp35S	hCas9	*WI12$_{Rhg1}$*; *DELLA11* and *18*	EGFP
	[37]			pFGC5941	pGmU6	pGmUbi; p35S	LbCpf1; ttLbCpf1	*FAD2-A* (*Glyma10g42470)*; *MCT* (*Glyma10G276600*); *RPL15* (*Glyma10G273900*); *PDCD6IP* (*Glyma10G266600*)	Bar
	[38]			pCAMBIA3301 based vectors	pGmU6-10	p35S	pCas9	*GmNAC06*	Blp
	[39]			GoldenGate GATEWAY	p35S	p35S	Cas9 fused with Csy4 via P2A peptide	*GW2*	Bar
	[40]			pCAMBIA3301 based vectors	pGmU6	p35S	pCas9	*GmCRY1a-1d; 2a-2b*	Blp
	[41]			pCAMBIA3301 based vector[PC]	pGmU6-6[PC]	2xp35S[PC]	Cas9[PC]	*lncRNA77580*	Blp
	[42]			pBSE401 [17]	pAtU6-26; pAtU6-29	p35S	zCas9	*GmNHX5*	Blp; **PCR**
	[43]			not reported	not reported	not reported	Cas9	*galactinol synthase* genes (G03 and 19)	Bar
	[44]			pCAMBIA3301 based vectors	pAtU6-26; pGmU6-10	p35S	Cas9	*GmpPLA-IIε*; *GmpPLA-IIζ*	Blp; **PCR**

Plant species	Reference	Year	Agrobacterium strain	Vector name or type of cloning system	Promoters for gRNA expression	Promoters for Cas9 expression	Cas9 type	Gene chosen for genome editing (name or ID)	Marker of transgenic hairy roots
	[45]			JRH0951 (gRNA); JRH0945 (Cas9)	pGmU6-10	2xp35S	Cas9	*GmCONSTANS*	Bar
	[46]			custom modified pCAMBIA1300 based vectors	pGmU6	pM4	Cas9	*miR169c*	Bar
	[47]			pSoy10	not reported	not reported	Cas9	*GmDDR1*	DsRed2
***Glycine soja* (wild soybean)**	[48]	2020	K599	pCAMBIA3301 based vectors	pGmU6-10	p35S	Cas9	*SOS1* (plasma membrane Na^+/H^+ antiporter); nonselective cation channels *(NSCC)*	Blp
***Glycyrrhiza glabra* (liquorice)**	[49]	2021	ATCC15834	pHSE401 [17]	pAtU6-26	2xp35S	zCas9	*F5H*	Hygr
***Glycyrrhiza uralensis* (Chinese liquorice)**	[50]	2021	ATCC15834	pHSE401 [17]	pAtU6-26	2xp35S	zCas9	*β-AS*	Hygr
***Gossypium hirsutum* (cotton)**	[51]	2021	K599	custom modified pCAMBIA2301	pAtU6-26	2xp35S	Cas9	*GhMYB25-like A* and *D*	Km
Lotus japonicus	[52]	2016	LBA1334*	custom modified pCAMBIA1300	pLjU6-1	2xp35S; pLjLb2	Cas9	*Leghemoglobins* (*Lb*) *b1*; *b2* and *b3*	sGFP
	[53]	2019	ATCC15834	pMgP237-2A-GFP [5]	pAtU6-26	2xp35SΩ	AtCas9 fused with GFP via T2A peptide	*CYP716A51*	**GFP**; Km
***Lupinus albus* (white lupin)**	[54,55]	2020	A4T	pCas9::ALMT-sgRNA1/2	pAtU6-6^{PC}	pPcUbi4-2^{PC}	pCas9	*LaALMT1* (*(Al)-activated malate transporter 1*)	Bar; **GFP**
	[54,56]	2021		pCas9::MATEPS1; pCas9::MATEPS2				*LaMATE*; *LaMATE3* (*Multidrug and Toxic Compound Extrusion*)	
Medicago truncatula	[13]	2015	ARqua1*	pMDC32/GmCas9	pAtU6-26	2xp35S	GmCas9	*GUS* gene in *GUS* barrelclover line	Hygr

Plant species	Reference	Year	Agrobacterium strain	Vector name or type of cloning system	Promoters for gRNA expression	Promoters for Cas9 expression	Cas9 type	Gene chosen for genome editing (name or ID)	Marker of transgenic hairy roots
	[57]	2017		custom modified pKSE401 [17]	pAtU6-26	2xp35S	zCas9	*DZA315(Fix*$^{+}$*) allele of Medtr8g465280* (*NFS2*)	**GUS**; Km
	[58]							*NCR-α; NCR-β*	
	[59]	2018						*NFS*$^{1-}$; *NFS*$^{2-}$ alleles	
	[60]	2020	LBA9402	pYLCRISPR/Cas9 P35s-B [10]	pAtU3b	2xp35S	pCas9 with highest GC content at the 5′ terminal region for Gramineae genes	*PDS*	Bar
Medicago truncatula	[61]	2020	ARqua1*	pHSN401 [17]	pAtU6-26; pMtU6-1	2xp35S	zCas9	*MtCRA2; MtEIN2*	Hygr
***Nicotiana tabacum* (tobacco)**	[62]	2020	ATCC15834	GoldenBraid 4.0	*snoRNA* promoters	p35S	hCas9	*N-methylputrescine oxidase* (*MPO*) gene family	**Km**; **DsRed**
Ophiorrhiza pumila	[63]	2020	C58C1*	pCAMBIA1300 based vectors	pAtU6	pAtUbi	hCas9	*secologanin synthetase; geraniol-10-hydroxylase*	Hygr
Phtheirospermum japonicum	[64]	2021	AR1193*	GoldenGate	pAtU6	2xp35S	hCas9	*PjIPT1a*	DsRed
***Populus tremula* x *alba* (hybrid polar)**	[65]	2020	ARqua1*	p201G/Cas9; pUC-gRNA [2]	pMtU6.6	2xp35S	hCas9	*UDP-dependent glycosyltransferases* (*UGT71L1, UGT78M1*)	EGFP
	[66]	2021		MoClo system	pAtU6-26; pAtU6-29	pAtAct2	Cas9	*SHORTROOT; YUC4; PLETHORA1; LBD4; LBD12*	tdTomato
***Punica granatum* (pomegranate)**	[67]	2019	MSU440*	custom modified pCAMBIA1300	pAtU6	pAtUbi1	Cas9	*UDP-dependent glycosyltransferases* (*UDP84A23; UDP84A24*)	GFP
***Salvia miltiorrhiza* (red sage)**	[68]	2017	ACCC10060	pCAMBIA1300 based vectors	pAtU6-26	2xp35S	Cas9	*committed diterpene synthase 1*	Hygr

Plant species	Reference	Year	Agrobacterium strain	Vector name or type of cloning system	Promoters for gRNA expression	Promoters for Cas9 expression	Cas9 type	Gene chosen for genome editing (name or ID)	Marker of transgenic hairy roots
	[69]	2018	C58C1*		pAtU6-26; pOsU3	pAtUbi; pOsUbi	hCas9	*rosmarinic acid synthase*	
	[70] [71]	2020	C58C1 (pRiA4)*		pAtU6	pAtUbi	hCas9	*bZIP1* *MYB98*	
	[72]	2021	ATCC15834	pICSL002218A (TSL SynBio, Norwich, UK)	pAtU6-26[PC]	pAtUbi10[PC]	hCas9[PC]	*SmKFB5*	**Km**; **PCR**[PC]
	[73]	2021	C58C1*	pCAMBIA1300 based vectors	pAtU6	pAtUbi	Cas9	23 *LACCASE* genes (*1-4*; *8-13*; *15-24*; *26-28*)	Hygr
***Solanum lycopersicum* (tomato)**	[74]	2014	ATCC15834	pMR093	pAtU6	p35S	*Nicotiana* sp. codon optimized Cas9	*GFP* and *SHR* genes in *SlSCRpro:modified (m) mGFP5* tomato line	BASTA
	[75]	2016	ARqua1*	p201N/Cas9 pUC-gRNA [2]	pMtU6.6	2xp35S	hCas9	no genes were reported	Km
***Solanum lycopersicum* (tomato)**	[76]	2019	ATCC15834	pMgP237-2A-GFP [5]	pAtU6-26	2xp35SΩ	AtCas9 fused with GFP via T2A peptide	*Sl5αR1*; *R2*	GFP; Km; **PCR**
	[77]	2020	ATCC15834	GoldenGate; pDe-Cas9(VQR)-Km; pDe-Cas9-Km	pAtU6	pPcUbi4-2	Cas9; Cas9(VQR)	*MYC1*; *MYC2*; *COI1*; *C5-SD2*	Km
***Solanum tuberosum* (potato)**	[5]	2018	ATCC15834	pEgP237-2A-GFP [78]	pAtU6-26	2xp35SΩ	AtCas9 fused with GFP via T2A peptide	*St16DOX* (*2-oxoglutarate-dependent dioxygenase*)	GFP; Km; **PCR**
	[79]	2020	MSU440*	GoldenGate cloning system and vectors developed by Čermák et al., 2017 [80]	pAtU6; pAt7SL	p35S	AtCas9; AtCas9 or AtCas9D10A both fused with dpCsy4 via P2A peptide	*PDS*	Hygr
	[81]	2020	15834 or 43056*	pBIN-HcoCas9: mGFP; pChimera (for gRNA construction)	pAtU6-26	2xp35S	hCas9	*GFP* gene in *GFP* potato line; *nonexpresser of pathogenesis-related 3*	mGFP

Plant species	Reference	Year	Agrobacterium strain	Vector name or type of cloning system	Promoters for gRNA expression	Promoters for Cas9 expression	Cas9 type	Gene chosen for genome editing (name or ID)	Marker of transgenic hairy roots
***Symphytum officinale* (comfrey)**	[82]	2021	ATCC15834	pEn-Chimera (for sgRNA); modified pDe-Cas9 [83]	pAtU6-26	pPcUbi4-2	AtCas9	*homospermidine synthase*	Hygr
***Taraxacum kok-saghyz* (rubber dandelion)**	[84]	2016	K599	pFGC-pcoCas9 (AG # 52256); pICH86966 (gRNA) [85]	pAtU6	p35SPPDK	pCas9	*fructan:fructan 1-fructosyltransferase*	BASTA; **PCR**
***Vigna unguiculata* (cowpea)**	[86]	2019	K599	custom modified pCAMBIA1300	pLjU6-1	2xp35S	Cas9	*VuSYMRK*	sGFP

***Agrobacterium* strains:** * — *Agrobacterium rhizogenes* strains with the chromosomal background of *Agrobacterium tumefaciens* C58.

Cas9 types: AtCas9 — Arabidopsis codon optimized Cas9; **AtCas9D10A** — Arabidopsis codon optimized nickase Cas9D10A; **BnCas9** — rapeseed codon optimized Cas9; **Cas9(VQR)** — mutant Cas9 recognizing the 5′—NGA—3′ protospacer adjacent motif (PAM) instead of the 5′—NGG—3′ PAM; **dpCas9** — Cas9 codon optimized for dicots; **GmCas9** — soybean codon optimized Cas9; **hCas9** — human codon optimized Cas9; **Cpf1** — Cas nuclease from CRISPR system of *Prevotella* and *Francisella* 1 (also known as Cas12); **LbCpf1** —Cpf1 nuclease of *Lachnospiraceae* bacterium (recognizing the 5′—TTTN—3′ PAM instead of the NGG PAM); **ttLbCpf1** — temperature-tolerant LbCpf1 variant; **pCas9** — plant codon optimized Cas9; **zCas9** — maize codon optimized Cas9; izCas9 — zCas9 with multiple introns.

Promoters: **pAct2** — promoter of the *actin2* gene; **pLb** — leghemoglobin promoter**; p35S** — 35S promoter from the Cauliflower Mosaic Virus; **2xp35S** — double p35S; **2xp35SΩ** — 2xp35S with the omega translational enhancer from the Cauliflower Mosaic Virus 5′ UnTranslated Region (5′-UTR); **p35SPPDK** — constitutive *35S* enhancer fused to the maize C4 pyruvate orthophosphate dikinase (C4PPDK) basal promoter**; pM4** — promoter of the soybean *SCREAM M4* gene; **pUbi** — promoter of a *ubiquitin* gene; **pU6**, **pU3** or **p7SL** — promoters of the small nucleolar RNA (*snoRNA*) genes.

Markers for transgenic hairy root detection: Bar (synonyms **BASTA, Blp** or **PPT**) — gene encoding resistance to the phosphinothricin (PPT) or to the PPT-containing compounds (e.g. tripeptide bialaphos (Blp), herbicide BASTA®); **DsRed** — Red Fluorescent Protein from *Discosoma* sp.; **GFP** — green fluorescent protein from *Aequorea victoria* and it variants: **3xGFP** — triple GFP, **EGFP** — enhanced GFP, **mGFP** — monomeric GFP, **sGFP** — synthetic GFP; **GUS** — *β*-glucuronidase; **GUS/GFP** — bifunctional fusion between GUS and GFP; **Hygr** — hygromycin resistance; **Km** — kanamycin resistance; **MYB75/PAP1** — anthocyanin pigmentation screenable marker encoded by the *MYELOBLASTOSIS75/PRODUCTION OF ANTHOCYANIN PIGMENTS1* (*MYB75/PAP1*) gene; **PCR** — genotyping by the polymerase chain reaction (PCR); **tdTOMATO** — tandem linked dimers of the Red Fluorescent Protein TOMATO (DsRed1 derivative). If more than one selectable/screenable marker was present in the CRISPR/Cas9 plasmid, the marker preferred by investigators is listed in underlined bold print.

Species abbreviations: At — *Arabidopsis thaliana*; **Ci** — *Cichorium intybus*; **Gm** — *Glycine max*; **La** — *Lupinus albus*; **Lj** — *Lotus japonicus*; **Mt** — *Medicago truncatula*; **Os** — *Oryza sativa*; **Pc** — *Petroselinum crispum*; **Sl** — *Solanum lycopersicum*; **Zm** — *Zea mays*.

Other vector components and abbreviations: ADH — translational enhancer from the 5′-UTR of the *alcohol dehydrogenase* gene; **AG** — AddGene (plasmid repository); **Csy4** — RNA endoribonuclease Csy4 from *Pseudomonas aeruginosa***; dpCsy4** — dicots codon optimized Csy4; **PC** — personal communication (used when the information about *A. rhizogenes* strain or vector components was not found in a published study, either in the main text or in the supplementary materials; PC was used for refs 4, 8, 39; 49-51, 67); **P2A** — self cleaving 2A peptide from porcine teschovirus-1 (PTV); **T2A** — self cleaving 2A peptide from *Thosea assigna* virus (TaV).

Table S2. Examples of the old-known conventional and nonconventional markers used for identification of transgenicity in transformed plants

Year of the first report about marker usage for identification of transgenicity	Reference	Plant species chosen for testing the marker	Common name of marker	Type of marker	Protein	Source of marker (including year and reference)
**1983**	[87]	*Nicotiana tabacum*	Neo/Km	antibiotic resistance (selectable)	neomycin phosphotransferase II (NPTII) or aminoglycoside 3-phosphotransferase II (APH (3′)-II)	*nptII* gene from the Tn*5* transposon (1982) [88]
			Mtx		methotrexate-insensitive dihydrofolate reductase (DHFR)	*dhfr* gene from the *Tn7* transposon (1982) [89]
**1985 (March)***	[90]	*Nicotiana tabacum*	Hygr	antibiotic resistance (selectable)	hygromycin phosphotransferase (HPT)	*Escherichia coli aphIV* gene (1983) [91,92]
**1985 (October)**	[93]	*Nicotiana tabacum*	Glp	herbicide resistance (to *N*-phosphono methylglycin (glyphosate) containing herbicides, e.g. Roundup®) (selectable)	5-enolpyruvyl-shikimate 3-phosphate (EPSP) synthase	*Salmonella tiphimurium* mutant allele of the *aroA* gene (1983) [94]
**1986**	[95]	*Nicotiana plumbaginifolia*	Bm	antibiotic resistance (selectable)	glycopeptide interacting with DNA causes double strand breaks	*ble* gene from the Tn*5* transposon (1985) [96,97]
**1987 (September)**	[98]	*Nicotiana tabacum*<br>*Solanum lycopersicum*<br>*Solanum tuberosum*	Bar (Blp; BASTA)	herbicide resistance (to phosphinothricin (PPT) or bialaphos containing herbicides, e.g. BASTA®) (selectable)	phosphinothricin *N*-acetyltransferase (PAT)	*Streptomyces hygroscopicus bar* gene conferring resistance to bialaphos (the tripeptide containing PPT) (1986) [99]
**1987 (November)**	[100]	*Nicotiana tabacum*<br>*Nicotiana plumbaginifolia*	Str (Sm)	antibiotic resistance (selectable)	streptomycin phosphotransferase (SPT)	*spt* gene from the Tn*5* transposon (1983/85) [97,101]
**1988 (February)**	[102]	*Arabidopsis thaliana*	ALS	herbicide resistance (to the sulfonylureas, e.g. chlorsulfuron) (selectable)	acetolactate synthase (ALS)	*als* gene from the chlorsulfuron resistant *Arabidopsis thaliana* mutant (1988) [102]
**1988 (April)**	[103]	*Arabidopsis thaliana*<br>*Petunia hybrida*	Gm/Neo/Km	antibiotic resistance (selectable)	aminoglycoside-3-*N*-acetyl transferases AAC(3)-III and IV	*aacC3* and *C4* genes from the IS*140* mobile element (1984/85) [104,105]

Year of the first report about marker usage for identification of transgenicity	Reference	Plant species chosen for testing the marker	Common name of marker	Type of marker	Protein	Source of marker (including year and reference)
**1988 (May)**	[106]	*Zea mays*	GUS	colorimetric (screenable)	β-glucuronidase	*Escherichia coli* *uidA* gene (1973/86) [107,108]
**1989 (August)**	[109]	*Nicotiana tabacum*	2,4-D	2,4-dichlorophenoxyacetate (synthetic auxin) resistance (selectable)	2,4-dichlorophenoxyacetate monooxygenase (DPAM)	the soil bacterium *Alcaligenes eutrophus* *tfdA* gene (1987) [110]
**1989 (October)**	[111]	*Nicotiana tabacum*	Pm/Bm	antibiotic resistance (selectable)	glycopeptide interacting with DNA causes double strand breaks	*ble* gene from the Tn*3* transposon (1985) [96,97]; the actinomycete *Streptoalloteichus hindustanus* *ble* gene (1990) [112]
**1990 (January)**	[113]	*Zea mays*	anthocyanin pigmentation	colorimetric (screenable)	transcription factor of the basic Helix-Loop-Helix (bHLH) family	*Zea maize* *Leaf color* gene (*ZmLc*) (*bHLH*) (1989) [114]
**1990 (February)**	[115]	*Nicotiana tabacum*	Str/Sp	antibiotic resistance (selectable)	aminoglycoside-(3") (9)-adenylyltransferase (AadA)	*Escherichia coli* *aadA* gene (1985) [116]
**1990 (September)**	[117]	*Nicotiana tabacum*	Bsd	antibiotic resistance (selectable)	blasticidin S deaminase (Bsd) (aminohydrolase)	*Bacillus cereus* *bsr* gene (1987/88) [118,119]
**1991**	[120]	*Nicotiana tabacum*	Gm	antibiotic resistance (selectable)	aminoglycoside-3-*N*-acetyltransferase (AAC(3)-I)	*aacC1* gene from the Tn*21*-like transposon [121]
**1995**	[122]	*Citrus sinensis*	GFP	fluorescent protein (screenable)	Green Fluorescent Protein	*gfp10* template obtained from the native *gfp* of the jellyfish *Aequorea victoria* (1992) [123]
**1998 (April)**	[124]	*Beta vulgaris*	ManA	selection for ability to metabolize mannose (selectable)	phosphomannose isomerase (PMI)	*Escherichia coli* *manA* gene (1984) [125]
**1998 (May)**	[126]	*Nicotiana tabacum* *Solanum lycopersicum* *Solanum tuberosum*	XylA	selection for ability to metabolize xylose (selectable)	xylose isomerase	*Clostridium thermosulfurogenes* *xylA* gene (1990) [127]
**2001**	[128]	*Nicotiana tabacum*	DsRed	fluorescent protein (screenable)	Orange (Red) Fluorescent Protein	*Discosoma* sp. *DsRed* gene (1999) [129]

Year of the first report about marker usage for identification of transgenicity	Reference	Plant species chosen for testing the marker	Common name of marker	Type of marker	Protein	Source of marker (including year and reference)
**2004**	[130]	*Arabidopsis thaliana*	TPS1	selection for insensitivity to the medium containing glucose in high concenration (selectable)	trehalose-6-phophate synthase (TPS)	*Arabidopsis thaliana* *TPS1* gene (1998) [131]

*If two or more studies were published per same year, then studies were ranged by month of publication.

Table S3. List of research tasks solved using CRISPR/Cas editing of different genes in hairy roots.

Plant species	Reference	Year	Gene chosen for genome editing (name or ID)	Method of hairy roots obtaining*	Aims of studies reporting only identification of genome editing events in hairy roots	Aims of studies using both identification of genome editing events and phenotyping of edited hairy roots
***Arachis hypogaea* (peanut)**	[1]	2019	*ahFAD2a*	CP	testing of genome editing capability in hairy roots	
	[3]	2020	*NFR1A1; A2; B1; B2; NFR5A; B*	CP		root nodule (RN) symbiosysis investigation (phenotyping for nodules formation)
***Atropa belladonna* (belladonna)**	[4]	2021	*pyrrolidine ketide synthase* (*PYKS*)	LE		metabolic engineering (phenotyping for changes of tropane alcaloids)
***Brassica carinata* (Abyssinian mustard)**	[6]	2017	*FASCICLIN-LIKE ARABINOGALACTAN PROTEIN 1*	CP		root development (phenotyping for root hairs formation)
***Brassica napus* (rapeseed)**	[7]	2020	*HVA22 homologue C; AGO1*(*miR168*); *TAO1*(*miR1885a*)	CE	testing of genome editing eviciency before stable transformation	
***Catharanthus roseus* (Madagascar periwinkle)**	[8]	2021	*Jasmonate-associated MYC2; Jasmonate-associated MYC3; Repressor of MYC2 targets 1*	SE	testing of genome editing capability in hairy roots	
***Cichorium intybus* (chicory)**	[9]	2019	*PDS*	LE	regeneration to the whole genome edited plant	
***Eucalyptus grandis* (flodded gum)**	[11]	2020	*Cinnamoyl-CoA reductase1; IAA9A*	CP		root development (phenotyping for lignin biosynthesis)
***Glycine max* (soybean)**	[12]	2015	*bar* gene in *bar* soybean line; *GmFEI1; GmFEI2; GmSHR*	HE and CE	testing of genome editing capability in hairy roots	
	[2]		*gfp* gene in *gfp* soybean line; *Glyma07g14530; 01gDDM1; 11gDDM1; miR1509; miR1514; MET1*	HE and CE		
***Glycine max* (soybean)**	[13]	2015	*glutamine synthase 1; chalcone-flavanone isomerase 20*	CE	testing of genome editing capability in hairy roots	
	[14]		*Glyma06g14180; Glyma08g02290; Glyma12g37050*	CP		
	[15]	2016	*PDS*	CE	testing of genome editing eviciency before stable transformation	

Plant species	Reference	Year	Gene chosen for genome editing (name or ID)	Method of hairy roots obtaining*	Aims of studies reporting only identification of genome editing events in hairy roots	Aims of studies using both identification of genome editing events and phenotyping of edited hairy roots
	[16]		*Glyma.01G165800; Glyma.01G165800-D*	CP		RN symbiosysis investigation (phenotyping for nodules formation)
	[18]	2017	*Rg1*	CP		
	[19]	2018	*GmMYB118*	CP		investigation of resistance to the abiotic stress conditions (phenotyping of sensitivity to the drought)
	[20]		*Nod Factor Receptor 1a*	CP		RN symbiosysis investigation (phenotyping for nodules formation)
	[21]	2019	*LATE ELONGATED HYPOCOTYL 1a; 1b; 2a; 2b*	CP	testing of genome editing eviciency before stable transformation	
	[22]		*GmFAD2-1A; GmFAD2-1B*	CP		
	[23]		*Glyma.03g163500; Glyma.10g037100; Glyma.10g246300; Glyma.13g123500; Glyma.19g164800; Glyma.19g164900; Glyma.20g146200; Glyma.20g148200; Glyma.20g148400*	CP	testing of genome editing capability in hairy roots	
	[24]		*Isoflavonoid syntase*	CE		metabolic engineering (phenotyping for changes in isoflavone metabolic pathway)
***Glycine max* (soybean)**	[25]	2020	74 nodulation genes; 27 seed genes	CP	testing of genome editing eviciency before stable transformation	
	[26]		*Rg1*	CP		RN symbiosysis investigation (phenotyping for nodules formation)
	[27]		*galactinol synthase* (*GOLS*) genes: *GOLS1A; GOLS1B*	CE	testing of genome editing eviciency before stable transformation	
	[29]		*GmPHR1; GmPHR4*	CP		RN symbiosysis investigation (phenotyping for nodules formation)
	[31]		*GmF3H1; GmF3H2; GmFNSII-1*	CE	testing of genome editing eviciency before stable transformation	
	[32]		*GmAGO7a; b*	CP		

Plant species	Reference	Year	Gene chosen for genome editing (name or ID)	Method of hairy roots obtaining*	Aims of studies reporting only identification of genome editing events in hairy roots	Aims of studies using both identification of genome editing events and phenotyping of edited hairy roots
	[33]		*Glyma15G191200*	CE		investigation of resistance to the biotic stress conditions (phenotyping for resistance to soybean cyst nematode)
	[34]		*GmIPK1*; *GmIPK2*		testing of genome editing capability in hairy roots	
	[35]		*Glyma06g14180*			
	[36]	2021	*DELLA 11 and 18*; *WI12$_{Rhg1}$*	CE		investigation of resistance to the biotic stress conditions (phenotyping of interaction with root knot nematode)
	[37]		*FAD2-A* (*Glyma10g42470*); *MCT* (*Glyma10G276600*); *RPL15* (*Glyma10G273900*); *PDCD6IP* (*Glyma10G266600*)		testing of genome editing capability in hairy roots	
	[38]		*GmNAC06*	CP		investigation of resistance to the abiotic stress conditions (phenotyping of sensitivity to the salinity)
**_Glycine max_ (soybean)**	[39]	2021	*GW2*	CE	testing of genome editing capability in hairy roots	
	[40]		*GmCRY1a-1d; 2a-2b*	CP	testing of genome editing eviciency before stable transformation	
	[41]		*lncRNA77580*	HE		investigation of resistance to the abiotic stress conditions (phenotyping for gene expression driven by salt stress)
	[42]		*GmNHX5*	CE		investigation of resistance to the abiotic stress conditions (phenotyping of sensitivity to the salinity)
	[43]		*galactinol synthase* genes (*G03* and *G19*)	CE	testing of genome editing capability in hairy roots	
	[44]		*GmpPLA-IIε; GmpPLA-IIζ*	CE	testing of genome editing eviciency before stable transformation	
	[45]		*GmCONSTANS*	CE	testing of genome editing capability in hairy roots	
	[46]		*miR169c*	CP	testing of genome editing eviciency before stable transformation	RN symbiosysis investigation (phenotyping for nodules formation)

Plant species	Reference	Year	Gene chosen for genome editing (name or ID)	Method of hairy roots obtaining*	Aims of studies reporting only identification of genome editing events in hairy roots	Aims of studies using both identification of genome editing events and phenotyping of edited hairy roots
	[47]		*GmDDR1*	not reported		investigation of resistance to the biotic stress conditions (phenotyping for resistance to *Phytophthora sojae*)
***Glycine soja* (wild soybean)**	[48]	2020	*SOS1* (plasma membrane Na^+/H^+ antiporter); nonselective cation channels *(NSCC)*	CP		root development (phenotyping for root to shoot Na^+ and K^+ transport)
***Glycyrrhiza glabra* (liquorice)**	[49]	2021	*F5H*	HE		metabolic engineering (phenotyping for changes of glycyrrhizin (glycyrrhizic acid) content)
***Glycyrrhiza uralensis* (Chinese liquorice)**	[50]	2021	*β-AS*	HE		
***Gossypium hirsutum* (cotton)**	[51]	2021	*GhMYB25-like A* and *D*	CP	testing of genome editing capability in hairy roots	
Lotus japonicus	[52]	2016	*Leghemoglobins* (*Lb*) *b1*; *b2* and *b3*	CP		RN symbiosysis investigation (phenotyping for nodules formation)
	[53]	2019	*CYP716A51*			metabolic engineering (phenotyping of changes in C-28 oxidized triterpenoid production)
***Lupinus albus* (white lupin)**	[54,55]	2020	*LaALMT1* ((*Al*)*-activated malate transporter 1*)	CP		root development (phenotyping for root to shoot transport: characterization of xylem sap and shoot metal concentrations
	[54,56]	2021	*LaMATE*; *LaMATE3* (*Multidrug and Toxic Compound Extrusion*)	CP		root development (evaluation of citrate secretion from roots under low phosphorus conditions) metabolic engineering (phenotyping for changes of isoflavonoids or their glycosides content)
Medicago truncatula	[13]	2015	*GUS* gene in *GUS* barrelclover line	CP	testing of genome editing capability in hairy roots	
	[57]	2017	*DZA315(Fix⁺) allele of Medtr8g465280* (*NFS2*)	CP		RN symbiosysis investigation (phenotyping for nodules formation)
	[58]		*NCR-α*; *NCR-β*	CP		RN symbiosysis investigation (phenotyping for nodules formation)
	[59]	2018	*NFS1⁻*; *NFS2⁻* alleles			

Plant species	Reference	Year	Gene chosen for genome editing (name or ID)	Method of hairy roots obtaining*	Aims of studies reporting only identification of genome editing events in hairy roots	Aims of studies using both identification of genome editing events and phenotyping of edited hairy roots
	[60]	2020	*PDS*	HE	regeneration to the whole genome edited plant	
	[61]		*MtCRA2*; *MtEIN2*	CP		RN symbiosysis investigation (phenotyping for Rhizobial early infection events)
Nicotiana tabacum **(tobacco)**	[62]	2020	*N-methylputrescine oxidase* (*MPO*) gene family	LE	regeneration to the whole genome edited plant	
Ophiorrhiza pumila	[63]	2020	*secologanin synthetase*; *geraniol-10-hydroxylase*	SE		metabolic engineering (phenotyping for changes of camptothecin production)
Phtheirospermum japonicum	[64]	2021	*PjIPT1a*	CP		investigation of resistance to the biotic stress conditions (phenotyping of interaction between hemiparasitic plant *P. japonicum* and Arabidopsis)
Populus tremula* x *alba **(hybrid polar)**	[65]	2020	*UDP-dependent glycosyltransferases* (*UGT71L1*, *UGT78M1*)	LE		metabolic engineering (phenotyping for changes of salicinoid content)
	[66]	2021	*SHORTROOT*; *YUC4*; *PLETHORA1*; *LBD4*; *LBD12*	LE	testing of genome editing capability in hairy roots (for *YUC4*; *PLETHORA1*; *LBD4*; *LBD12*)	root development (phenotyping for endodermis development: for *SHORTROOT*)
Punica granatum **(pomegranate)**	[67]	2019	*UDP-dependent glycosyltransferases* (*UDP84A23*; *UDP84A24*)	HE		metabolic engineering (phenotyping for changes of gallic acid and its glicosides content)
Salvia miltiorrhiza **(red sage)**	[68]	2017	*committed diterpene synthase 1*	LE		metabolic engineering (phenotyping for changes of tashinones (Ts) content)
	[69]	2018	*rosmarinic acid synthase*			metabolic engineering (phenotyping for changes of lithospermic acid and its precursors)
	[70]	2020	*bZIP1*	LE		metabolic engineering (phenotyping for changes in phenolic acid and Ts content)
	[71]		*MYB98*			metabolic engineering (phenotyping for changes in salvinalonic acid B (SAB) and Ts content)

Plant species	Reference	Year	Gene chosen for genome editing (name or ID)	Method of hairy roots obtaining*	Aims of studies reporting only identification of genome editing events in hairy roots	Aims of studies using both identification of genome editing events and phenotyping of edited hairy roots
	[72]	2021	*SmKFB5*			metabolic engineering (phenotyping for changes in SAB, rosmarinic (RA) and caffeic acids content)
	[73]		23 *LACCASE* genes (*1-4*; *8-13*; *15-24*; *26-28*)			metabolic engineering (phenotyping for changes in SAB, RA content; lignin biosynthesis pathway)
***Solanum lycopersicum* (tomato)**	[74]	2014	*GFP* and *SHR* genes in *SlSCRpro:modified (m) mGFP5* tomato line	CP	testing of genome editing capability in hairy roots (for *GFP*)	root development (phenotyping for meristem size: for *SHR*)
	[75]	2016	no genes were reported	CE	testing of genome editing capability in hairy roots	
	[76]	2019	*Sl5αR1*; *R2*	HE		metabolic engineering (phenotyping for changes in α-tomatine and dehydrotomatine biosynthesis pathway)
	[77]	2020	*MYC1*; *MYC2*; *COI1*; *C5-SD2*	CE		metabolic engineering (phenotyping for changes in α-tomatine and dehydrotomatine biosynthesis via supression of cholesreol and steroidal glycoalkaloid biosynthesis genes)
***Solanum tuberosum* (potato)**	[5]	2018	*St16DOX* (*2-oxoglutarate-dependent dioxygenase*)	CP		metabolic engineering (phenotyping for changes in α-solanine and α-chaconine biosynthesis pathway)
	[79]	2020	*PDS*	SE	regeneration to the whole genome edited plant	
	[81]		*GFP* gene in *GFP* potato line; *nonexpresser of pathogenesis-related 3*	CP	testing of genome editing capability in hairy roots (for *GFP*)	investigation of resistance to the biotic stress conditions (phenotyping of tolerance to *Candidatus* Liberibacter solanacearum)
***Symphytum officinale* (comfrey)**	[82]	2021	*homospermidine synthase*	LE		metabolic engineering (phenotyping for changes of honospermydine content)

Plant species	Reference	Year	Gene chosen for genome editing (name or ID)	Method of hairy roots obtaining*	Aims of studies reporting only identification of genome editing events in hairy roots	Aims of studies using both identification of genome editing events and phenotyping of edited hairy roots
Taraxacum kok-saghyz **(rubber dandelion)**	[84]	2016	*fructan:fructan 1-fructosyltransferase*	CP	regeneration to the whole genome edited plant	
Vigna unguiculata **(cowpea)**	[86]	2019	*VuSYMRK*	CP		RN symbiosysis investigation (phenotyping for nodules formation)

*Hairy roots were obtained from composite plants (CP); cotyledon explants (CE); hypocotyl explants (HE); leaf explants (LE) or stem explants (SE).

Supplementary references

1. Yuan, M.; Zhu, J.; Gong, L.; He, L.; Lee, C.; Han, S.; Chen, C.; He, G. Mutagenesis of *FAD2* genes in peanut with CRISPR/Cas9 based gene editing. *BMC Biotechnol.* **2019**, *19*, 1-7, doi:10.1186/s12896-019-0516-8.
2. Jacobs, T.B.; LaFayette, P.R.; Schmitz, R.J.; Parrott, W.A. Targeted genome modifications in soybean with CRISPR/Cas9. *BMC Biotechnol.* **2015**, *15*, 1-10, doi:10.1186/s12896-015-0131-2.
3. Shu, H.; Luo, Z.; Peng, Z.; Wang, J. The application of CRISPR/Cas9 in hairy roots to explore the functions of *AhNFR1* and *AhNFR5* genes during peanut nodulation. *BMC Plant Biol.* **2020**, *20*, 1-15, doi:10.1186/s12870-020-02614-x.
4. Hasebe, F.; Yuba, H.; Hashimoto, T.; Saito, K.; Funa, N.; Shoji, T. CRISPR/Cas9-mediated disruption of the *PYRROLIDINE KETIDE SYNTHASE* gene reduces the accumulation of tropane alkaloids in *Atropa belladonna* hairy roots. *Biosci., Biotechnol., Biochem.* **2021**, *85*, 2404-2409, doi:10.1093/bbb/zbab165.
5. Nakayasu, M.; Akiyama, R.; Lee, H.J.; Osakabe, K.; Osakabe, Y.; Watanabe, B.; Sugimoto, Y.; Umemoto, N.; Saito, K.; Muranaka, T., et al. Generation of α-solanine-free hairy roots of potato by CRISPR/Cas9 mediated genome editing of the *St16DOX* gene. *Plant Physiol. Biochem.* **2018**, *131*, 70-77, doi:10.1016/j.plaphy.2018.04.026.
6. Kirchner, T.W.; Niehaus, M.; Debener, T.; Schenk, M.K.; Herde, M. Efficient generation of mutations mediated by CRISPR/Cas9 in the hairy root transformation system of *Brassica carinata*. *PLOS ONE* **2017**, *12*, 1-20, doi:10.1371/journal.pone.0185429.
7. Pröbsting, M. Application of CRISPR-Cas9 genome editing systems for improving oilseed rape (*Brassica napus*) disease resistance against *Verticillium longisporum*. PhD thesis, Kiel University, 2020.
8. Grützner, R.; Martin, P.; Horn, C.; Mortensen, S.; Cram, E.J.; Lee-Parsons, C.W.T.; Stuttmann, J.; Marillonnet, S. High-efficiency genome editing in plants mediated by a Cas9 gene containing multiple introns. *Plant Commun.* **2021**, *2*, 1-15, doi:10.1016/j.xplc.2020.100135.
9. Bernard, G.; Gagneul, D.; Alves Dos Santos, H.; Etienne, A.; Hilbert, J.-L.; Rambaud, C. Efficient genome editing using CRISPR/Cas9 technology in Chicory. *Int. J. Mol. Sci.* **2019**, *20*, 1-18, doi:10.3390/ijms20051155.
10. Ma, X.; Zhang, Q.; Zhu, Q.; Liu, W.; Chen, Y.; Qiu, R.; Wang, B.; Yang, Z.; Li, H.; Lin, Y., et al. A robust CRISPR/Cas9 system for convenient, high-efficiency multiplex genome editing in monocot and dicot plants. *Mol. Plant* **2015**, *8*, 1274-1284, doi:10.1016/j.molp.2015.04.007.
11. Dai, Y.; Hu, G.; Dupas, A.; Medina, L.; Blandels, N.; San Clemente, H.; Ladouce, N.; Badawi, M.; Hernandez-Raquet, G.; Mounet, F., et al. Implementing the CRISPR/Cas9 technology in Eucalyptus hairy roots using wood-related genes. *Int. J. Mol. Sci.* **2020**, *21*, 1-22, doi:10.3390/ijms21103408.
12. Cai, Y.; Chen, L.; Liu, X.; Sun, S.; Wu, C.; Jiang, B.; Han, T.; Hou, W. CRISPR/Cas9-mediated genome editing in soybean hairy roots. *PLOS ONE* **2015**, *10*, 1-13, doi:10.1371/journal.pone.0136064.
13. Michno, J.-M.; Wang, X.; Liu, J.; Curtin, S.J.; Kono, T.J.Y.; Stupar, R.M. CRISPR/Cas mutagenesis of soybean and *Medicago truncatula* using a new web-tool and a modified Cas9 enzyme. *GM Crops & Food* **2015**, *6*, 243-252, doi:10.1080/21645698.2015.1106063.
14. Sun, X.; Hu, Z.; Chen, R.; Jiang, Q.; Song, G.; Zhang, H.; Xi, Y. Targeted mutagenesis in soybean using the CRISPR-Cas9 system. *Sci. Rep.* **2015**, *5*, 1-10, doi:10.1038/srep10342.

15. Du, H.; Zeng, X.; Zhao, M.; Cui, X.; Wang, Q.; Yang, H.; Cheng, H.; Yu, D. Efficient targeted mutagenesis in soybean by TALENs and CRISPR/Cas9. *J. Biotechnol.* **2016**, *217*, 90-97, doi:10.1016/j.jbiotec.2015.11.005.
16. Tang, F.; Yang, S.; Liu, J.; Zhu, H. *Rj4*, a gene controlling nodulation specificity in soybeans, encodes a thaumatin-like protein but not the one previously reported. *Plant Physiol.* **2016**, *170*, 26-32, doi:10.1104/pp.15.01661.
17. Xing, H.-L.; Dong, L.; Wang, Z.-P.; Zhang, H.-Y.; Han, C.-Y.; Liu, B.; Wang, X.-C.; Chen, Q.-J. A CRISPR/Cas9 toolkit for multiplex genome editing in plants. *BMC Plant Biol.* **2014**, *14*, 1-12, doi:10.1186/s12870-014-0327-y.
18. Fan, Y.; Liu, J.; Lyu, S.; Wang, Q.; Yang, S.; Zhu, H. The soybean *Rfg1* gene restricts nodulation by *Sinorhizobium fredii* USDA193. *Front. Plant Sci.* **2017**, *8*, 1-9, doi:10.3389/fpls.2017.01548.
19. Du, Y.-T.; Zhao, M.-J.; Wang, C.-T.; Gao, Y.; Wang, Y.-X.; Liu, Y.-W.; Chen, M.; Chen, J.; Zhou, Y.-B.; Xu, Z.-S., et al. Identification and characterization of *GmMYB118* responses to drought and salt stress. *BMC Plant Biol.* **2018**, *18*, 1-18, doi:10.1186/s12870-018-1551-7.
20. Tang, T.; Yu, X.; Yang, H.; Gao, Q.; Ji, H.; Wang, Y.; Yan, G.; Peng, Y.; Luo, H.; Liu, K., et al. Development and validation of an effective CRISPR/Cas9 vector for efficiently isolating positive transformants and transgene-free mutants in a wide range of plant species. *Front. Plant Sci.* **2018**, *9*, 1-14, doi:10.3389/fpls.2018.01533.
21. Cheng, Q.; Dong, L.; Su, T.; Li, T.; Gan, Z.; Nan, H.; Lu, S.; Fang, C.; Kong, L.; Li, H., et al. CRISPR/Cas9-mediated targeted mutagenesis of *GmLHY* genes alters plant height and internode length in soybean. *BMC Plant Biol.* **2019**, *19*, 1-11, doi:10.1186/s12870-019-2145-8.
22. Do, P.T.; Nguyen, C.X.; Bui, H.T.; Tran, L.T.N.; Stacey, G.; Gillman, J.D.; Zhang, Z.J.; Stacey, M.G. Demonstration of highly efficient dual gRNA CRISPR/Cas9 editing of the homeologous GmFAD2–1A and GmFAD2–1B genes to yield a high oleic, low linoleic and α-linolenic acid phenotype in soybean. *BMC Plant Biol.* **2019**, *19*, 1-14, doi:10.1186/s12870-019-1906-8.
23. Li, C.; Nguyen, V.; Liu, J.; Fu, W.; Chen, C.; Yu, K.; Cui, Y. Mutagenesis of seed storage protein genes in soybean using CRISPR/Cas9. *BMC Res. Notes* **2019**, *12*, 1-7, doi:10.1186/s13104-019-4207-2.
24. Uchida, K. A study on isoflavonoid metabolism using CRISPR/Cas9 in soybean. *Soy Protein Research, Japan* **2019**, *22*, 152-156.
25. Bai, M.; Yuan, J.; Kuang, H.; Gong, P.; Li, S.; Zhang, Z.; Liu, B.; Sun, J.; Yang, M.; Yang, L., et al. Generation of a multiplex mutagenesis population via pooled CRISPR-Cas9 in soya bean. *Plant Biotechnol. J.* **2020**, *18*, 721-731, doi:10.1111/pbi.13239.
26. Fan, Y.; Wang, X.; Li, H.; Liu, S.; Jin, L.; Lyu, Y.; Shi, M.; Liu, S.; Yang, X.; Lyu, S. Anthocyanin, a novel and user-friendly reporter for convenient, non-destructive, low cost, directly visual selection of transgenic hairy roots in the study of rhizobia-legume symbiosis. *Plant Methods* **2020**, *16*, 1-8, doi:10.1186/s13007-020-00638-w.
27. Le, H.; Nguyen, N.H.; Ta, D.T.; Le, T.N.T.; Bui, T.P.; Le, N.T.; Nguyen, C.X.; Rolletschek, H.; Stacey, G.; Stacey, M.G., et al. CRISPR/Cas9-mediated knockout of galactinol synthase-encoding genes reduces raffinose family oligosaccharide levels in soybean seeds. *Front. Plant Sci.* **2020**, *11*, 1-13, doi:10.3389/fpls.2020.612942.
28. Li, J.-F.; Norville, J.E.; Aach, J.; McCormack, M.; Zhang, D.; Bush, J.; Church, G.M.; Sheen, J. Multiplex and homologous recombination–mediated genome editing in Arabidopsis and *Nicotiana benthamiana* using guide RNA and Cas9. *Nat. Biotechnol.* **2013**, *31*, 688-691, doi:10.1038/nbt.2654.

29. Lu, M.; Cheng, Z.; Zhang, X.-M.; Huang, P.; Fan, C.; Yu, G.; Chen, F.; Xu, K.; Chen, Q.; Miao, Y., et al. Spatial divergence of *PHR-PHT1* modules maintains phosphorus homeostasis in soybean nodules. *Plant Physiol.* **2020**, *184*, 236-250, doi:10.1104/pp.19.01209.
30. Lin, M.-H.; Gresshoff, P.M.; Indrasumunar, A.; Ferguson, B.J. pHairyRed: a novel binary vector containing the DsRed2 reporter gene for visual selection of transgenic hairy roots. *Mol. Plant* **2011**, *4*, 537-545, doi:10.1093/mp/ssq084.
31. Zhang, P.; Du, H.; Wang, J.; Pu, Y.; Yang, C.; Yan, R.; Yang, H.; Cheng, H.; Yu, D. Multiplex CRISPR/Cas9-mediated metabolic engineering increases soya bean isoflavone content and resistance to soya bean mosaic virus. *Plant Biotechnol. J.* **2020**, *18*, 1384-1395, doi:10.1111/pbi.13302.
32. Zheng, N.; Li, T.; Dittman, J.D.; Su, J.; Li, R.; Gassmann, W.; Peng, D.; Whitham, S.A.; Liu, S.; Yang, B. CRISPR/Cas9-based gene editing using egg cell-specific promoters in Arabidopsis and soybean. *Front. Plant Sci.* **2020**, *11*, 1-15, doi:10.3389/fpls.2020.00800.
33. Butler, K.J.; Fliege, C.; Zapotocny, R.; Diers, B.; Hudson, M.; Bent, A.F. Soybean cyst nematode resistance quantitative trait locus *cqSCN-006* alters the expression of a γ-SNAP protein. *Mol. Plant-Microbe Interact.* **2021**, *MPMI07210163R*, 1-13, doi:10.1094/mpmi-07-21-0163-r.
34. Carrijo, J.; Illa-Berenguer, E.; LaFayette, P.; Torres, N.; Aragão, F.J.L.; Parrott, W.; Vianna, G.R. Two efficient CRISPR/Cas9 systems for gene editing in soybean. *Transgenic Res.* **2021**, *30*, 239-249, doi:10.1007/s11248-021-00246-x.
35. Cheng, Y.; Wang, X.; Cao, L.; Ji, J.; Liu, T.; Duan, K. Highly efficient *Agrobacterium rhizogenes*-mediated hairy root transformation for gene functional and gene editing analysis in soybean. *Plant Methods* **2021**, *17*, 1-12, doi:10.1186/s13007-021-00778-7.
36. Dong, J.; Hudson, M.E. $WI12_{Rhg1}$ interacts with DELLAs and mediates soybean cyst nematode resistance through hormone pathways. *Plant Biotechnol. J.* **2021**, *pbi.13709*, 1-14, doi:10.1111/pbi.13709.
37. Duan, K.; Cheng, Y.; Ji, J.; Wang, C.; Wei, Y.; Wang, Y. Large chromosomal segment deletions by CRISPR/LbCpf1-mediated multiplex gene editing in soybean. *J. Integr. Plant Biol.* **2021**, *63*, 1620-1631, doi:10.1111/jipb.13158.
38. Li, M.; Chen, R.; Jiang, Q.; Sun, X.; Zhang, H.; Hu, Z. GmNAC06, a NAC domain transcription factor enhances salt stress tolerance in soybean. *Plant Mol. Biol.* **2021**, *105*, 333-345, doi:10.1007/s11103-020-01091-y.
39. Luo, Y.; Na, R.; Nowak, J.S.; Qiu, Y.; Lu, Q.S.; Yang, C.; Marsolais, F.; Tian, L. Development of a Csy4-processed guide RNA delivery system with soybean-infecting virus ALSV for genome editing. *BMC Plant Biol.* **2021**, *21*, 1-12, doi:10.1186/s12870-021-03138-8.
40. Lyu, X.; Cheng, Q.; Qin, C.; Li, Y.; Xu, X.; Ji, R.; Mu, R.; Li, H.; Zhao, T.; Liu, J., et al. GmCRY1s modulate gibberellin metabolism to regulate soybean shade avoidance in response to reduced blue light. *Mol. Plant* **2021**, *14*, 298-314, doi:10.1016/j.molp.2020.11.016.
41. Niu, F.; Jiang, Q.; Sun, X.; Hu, Z.; Wang, L.; Zhang, H. Large DNA fragment deletion in *lncRNA77580* regulates neighboring gene expression in soybean (*Glycine max*). *Funct. Plant Biol.* **2021**, *48*, 1139-1147, doi:10.1071/FP20400.
42. Sun, T.; Ma, N.; Wang, C.; Fan, H.; Wang, M.; Zhang, J.; Cao, J.; Wang, D. A Golgi-localized sodium/hydrogen exchanger positively regulates salt tolerance by maintaining higher K^+/Na^+ ratio in soybean. *Front. Plant Sci.* **2021**, *12*, 1-15, doi:10.3389/fpls.2021.638340.

43. Thao, L.T.N.; Nhung, N.H.; Huy, L.Q.; Thao, B.P.; Ngoc, L.T.; Ngoc, P.B.; Ha, C.H.; Phat, D.T. Development of an *in vitro* hairy root induction system in different soybean cultivars for gene expression and genome editing studies. *Vietnam Journal of Biotechnology* **2021**, *19*, 459-470, doi:10.15625/1811-4989/15421.
44. Xiao, Y.; Karikari, B.; Wang, L.; Chang, F.; Zhao, T. Structure characterization and potential role of soybean phospholipases A multigene family in response to multiple abiotic stress uncovered by CRISPR/Cas9 technology. *Environ. Exp. Bot.* **2021**, *188*, 1-14, doi:10.1016/j.envexpbot.2021.104521.
45. Xiong, Y.-P.; Xu, C.-J.; Jin-Ming, S.; Zhao, L. Detection of target sites of CRISPR/Cas9 system in soybean mediated by *Agrobacterium rhizogenes*. *Chinese Journal of Oil Crop Sciences* **2021**, *43*, 161-170, doi:10.19802/j.issn.1007-9084.2020337.
46. Xu, H.; Li, Y.; Zhang, K.; Li, M.; Fu, S.; Tian, Y.; Qin, T.; Li, X.; Zhong, Y.; Liao, H. *miR169c-NFYA-C-ENOD40* modulates nitrogen inhibitory effects in soybean nodulation. *New Phytol.* **2021**, *229*, 3377-3392, doi:10.1111/nph.17115.
47. Yu, G.; Zou, J.; Wang, J.; Zhu, R.; Qi, Z.; Jiang, H.; Hu, Z.; Yang, M.; Zhao, Y.; Wu, X., et al. A soybean NAC homolog contributes to resistance to *Phytophthora sojae* mediated by dirigent proteins. *Crop J.* **2021**, *In press (corrected proof)*, 1-10, doi:10.1016/j.cj.2021.08.009.
48. Niu, F.; Jiang, Q.; Cheng, R.; Sun, X.; Hu, Z.; Wang, L.; Zhang, H. CRISPR/Cas9-mediated targeted mutagenesis of wild soybean (*Glycine soja*) hairy roots altered the transcription profile of the mutant. *J. Agric. Sci.* **2020**, *12*, 14-25, doi:10.5539/jas.v12n9p14
49. Zhang, Z.; Wang, D.; Yang, L.; Tian, S.; Yao, X.; Liu, Y. Ferulate 5-hydroxylase gene (*F5H*) regulation of glycyrrhizic acid biosynthesis determined by gene overexpression and knockout. *Acta Pharm. Sin.* **2021**, *56*, 1719-1726, doi:10.16438/j.0513-4870.2021-0196.
50. Wang, D.; Zhang, Z.; Yang, L.; Tian, S.; Liu, Y. *ARPI*, *β-AS*, and *UGE* regulate glycyrrhizin biosynthesis in *Glycyrrhiza uralensis* hairy roots. *Plant Cell Rep.* **2021**, *40*, 1285-1296, doi:10.1007/s00299-021-02712-6.
51. Lili, Z.; Yali, W.; Peilin, W.; Jiamin, W.; Hongmei, C. Detection of target sites edited in upland cotton by the CRISPR/Cas9 system mediated by *Agrobacterium rhizogenes*. *BMC Plant Biol.* **2021**, *In press (under review)*, doi:10.21203/rs.3.rs-1105254/v1.
52. Wang, L.; Wang, L.; Tan, Q.; Fan, Q.; Zhu, H.; Hong, Z.; Zhang, Z.; Duanmu, D. Efficient inactivation of symbiotic nitrogen fixation related genes in *Lotus japonicus* using CRISPR/Cas9. *Front. Plant Sci.* **2016**, *7*, 1-22, doi:10.3389/fpls.2016.01333.
53. Suzuki, H.; Fukushima, E.O.; Shimizu, Y.; Seki, H.; Fujisawa, Y.; Ishimoto, M.; Osakabe, K.; Osakabe, Y.; Muranaka, T. *Lotus japonicus* triterpenoid profile and characterization of the *CYP716A51* and *LjCYP93E1* genes involved in their biosynthesis *in planta*. *Plant Cell Physiol.* **2019**, *60*, 2496-2509, doi:10.1093/pcp/pcz145.
54. Zhou, Y. Functional characterization of genes involved in development and function of cluster roots in *Lupinus albus*. PhD thesis, University of Hohenheim, 2019.
55. Zhou, Y.; Neuhäuser, B.; Neumann, G.; Ludewig, U. LaALMT1 mediates malate release from phosphorus-deficient white lupin root tips and metal root to shoot translocation. *Plant Cell and Environ.* **2020**, *43*, 1691-1706, doi:10.1111/pce.13762.
56. Zhou, Y.; Olt, P.; Neuhäuser, B.; Moradtalab, N.; Bautista, W.; Uhde-Stone, C.; Neumann, G.; Ludewig, U. Loss of LaMATE impairs isoflavonoid release from cluster roots of phosphorus-deficient white lupin. *Physiol. Plant.* **2021**, *173*, 1207-1220, doi:10.1111/ppl.13515.

57. Wang, Q.; Yang, S.; Liu, J.; Terecskei, K.; Ábrahám, E.; Gombár, A.; Domonkos, Á.; Szűcs, A.; Körmöczi, P.; Wang, T., et al. Host-secreted antimicrobial peptide enforces symbiotic selectivity in *Medicago truncatula*. *Proc. Natl. Acad. Sci. U.S.A.* **2017**, *114*, 6854-6859, doi:10.1073/pnas.1700715114.
58. Yang, S.; Wang, Q.; Fedorova, E.; Liu, J.; Qin, Q.; Zheng, Q.; Price, P.A.; Pan, H.; Wang, D.; Griffitts, J.S., et al. Microsymbiont discrimination mediated by a host-secreted peptide in *Medicago truncatula*. *Proc. Natl. Acad. Sci. U.S.A.* **2017**, *114*, 6848-6853, doi:10.1073/pnas.1700460114.
59. Wang, Q.; Liu, J.; Li, H.; Yang, S.; Körmöczi, P.; Kereszt, A.; Zhu, H. Nodule-specific cysteine-rich peptides negatively regulate nitrogen-fixing symbiosis in a strain-specific manner in *Medicago truncatula*. *Mol. Plant-Microbe Interact.* **2018**, *31*, 240-248, doi:10.1094/mpmi-08-17-0207-r.
60. Zhang, H.; Cao, Y.; Zhang, H.; Xu, Y.; Zhou, C.; Liu, W.; Zhu, R.; Shang, C.; Li, J.; Shen, Z., et al. Efficient generation of CRISPR/Cas9-mediated homozygous/biallelic *Medicago truncatula* mutants using a hairy root system. *Front. Plant Sci.* **2020**, *11*, 1-11, doi:10.3389/fpls.2020.00294.
61. Zhu, F.; Deng, J.; Chen, H.; Liu, P.; Zheng, L.; Ye, Q.; Li, R.; Brault, M.; Wen, J.; Frugier, F., et al. A CEP peptide receptor-like kinase regulates auxin biosynthesis and ethylene signaling to coordinate root growth and symbiotic nodulation in *Medicago truncatula*. *Plant Cell* **2020**, *32*, 2855–2877, doi:10.1105/tpc.20.00248.
62. Vazquez-Vilar, M.; Garcia-Carpintero, V.; Selma, S.; Bernabé-Orts, J.M.; Sanchez-Vicente, J.; Salazar-Sarasua, B.; Ressa, A.; de Paola, C.; Ajenjo, M.; Fernández-del-Carmen, A., et al. Edition of complex gene families in tobacco with GoldenBraid 4.0, a multipurpose web-based platform for plant genome engineering. *bioRxiv* **2020**, *2020.10.06.327841*, doi:10.1101/2020.10.06.327841.
63. Shi, M.; Gong, H.; Cui, L.; Wang, Q.; Wang, C.; Wang, Y.; Kai, G. Targeted metabolic engineering of committed steps improves anti-cancer drug camptothecin production in *Ophiorrhiza pumila* hairy roots. *Ind. Crops Prod.* **2020**, *148*, 1-9, doi:10.1016/j.indcrop.2020.112277.
64. Greifenhagen, A.; Braunstein, I.; Pfannstiel, J.; Yoshida, S.; Shirasu, K.; Schaller, A.; Spallek, T. The *Phtheirospermum japonicum* isopentenyltransferase PjIPT1a regulates host cytokinin responses in *Arabidopsis*. *New Phytol.* **2021**, *232*, 1582-1590, doi:10.1111/nph.17615.
65. Fellenberg, C.; Corea, O.; Yan, L.-H.; Archinuk, F.; Piirtola, E.-M.; Gordon, H.; Reichelt, M.; Brandt, W.; Wulff, J.; Ehlting, J., et al. Discovery of salicyl benzoate UDP-glycosyltransferase, a central enzyme in poplar salicinoid phenolic glycoside biosynthesis. *Plant J.* **2020**, *102*, 99-115, doi:10.1111/tpj.14615.
66. Triozzi, P.; Schmidt, H.W.; Dervinis, C.; Kirst, M.; Conde, D. Simple, efficient and open-source CRISPR/Cas9 strategy for multi-site genome editing in *Populus tremula* × *alba*. *Tree Physiol.* **2021**, *41*, 2216-2227, doi:10.1093/treephys/tpab066.
67. Chang, L.; Wu, S.; Tian, L. Effective genome editing and identification of a regiospecific gallic acid 4-O-glycosyltransferase in pomegranate (*Punica granatum* L.). *Hortic. Res.* **2019**, *6*, 1-15, doi:10.1038/s41438-019-0206-7.
68. Li, B.; Cui, G.; Shen, G.; Zhan, Z.; Huang, L.; Chen, J.; Qi, X. Targeted mutagenesis in the medicinal plant *Salvia miltiorrhiza*. *Sci. Rep.* **2017**, *7*, 1-9, doi:10.1038/srep43320.
69. Zhou, Z.; Tan, H.; Li, Q.; Chen, J.; Gao, S.; Wang, Y.; Chen, W.; Zhang, L. CRISPR/Cas9-mediated efficient targeted mutagenesis of RAS in *Salvia miltiorrhiza*. *Phytochemistry* **2018**, *148*, 63-70, doi:10.1016/j.phytochem.2018.01.015.
70. Deng, C.; Shi, M.; Fu, R.; Zhang, Y.; Wang, Q.; Zhou, Y.; Wang, Y.; Ma, X.; Kai, G. ABA-responsive transcription factor bZIP1 is involved in modulating biosynthesis of phenolic acids and taABA-responsive transcription factor bZIP1 is involvednshinones in *Salvia miltiorrhiza*. *J. Exp. Bot.* **2020**, *71*, 5948-5962, doi:10.1093/jxb/eraa295.

71. Hao, X.; Pu, Z.; Cao, G.; You, D.; Zhou, Y.; Deng, C.; Shi, M.; Nile, S.H.; Wang, Y.; Zhou, W., et al. Tanshinone and salvianolic acid biosynthesis are regulated by *SmMYB98* in *Salvia miltiorrhiza* hairy roots. *J. Adv. Res.* **2020**, *23*, 1-12, doi:10.1016/j.jare.2020.01.012.
72. Yu, H.; Li, D.; Yang, D.; Xue, Z.; Li, J.; Xing, B.; Yan, K.; Han, R.; Liang, Z. SmKFB5 protein regulates phenolic acid biosynthesis by controlling the degradation of phenylalanine ammonia-lyase in *Salvia miltiorrhiza*. *J. Exp. Bot.* **2021**, *72*, 4915-4925, doi:10.1093/jxb/erab172.
73. Zhou, Z.; Li, Q.; Xiao, L.; Wang, Y.; Feng, J.; Bu, Q.; Xiao, Y.; Hao, K.; Guo, M.; Chen, W., et al. Multiplexed CRISPR/Cas9-mediated knockout of Laccase genes in *Salvia miltiorrhiza* revealed their roles in growth, development, and metabolism. *Front. Plant Sci.* **2021**, *12*, 1-10, doi:10.3389/fpls.2021.647768.
74. Ron, M.; Kajala, K.; Pauluzzi, G.; Wang, D.; Reynoso, M.A.; Zumstein, K.; Garcha, J.; Winte, S.; Masson, H.; Inagaki, S., et al. Hairy root transformation using *Agrobacterium rhizogenes* as a tool for exploring cell type-specific gene expression and function using tomato as a model. *Plant Physiol.* **2014**, *166*, 455-469, doi:10.1104/pp.114.239392.
75. Jacobs, T.B.; Martin, G.B. High-throughput CRISPR vector construction and characterization of DNA modifications by generation of tomato hairy roots. *J. Vis. Exp.* **2016**, *110*, 1-6, doi:10.3791/53843.
76. Akiyama, R.; Lee, H.J.; Nakayasu, M.; Osakabe, K.; Osakabe, Y.; Umemoto, N.; Saito, K.; Muranaka, T.; Sugimoto, Y.; Mizutani, M. Characterization of steroid 5α-reductase involved in α-tomatine biosynthesis in tomatoes. *Plant Biotechnol.* **2019**, *36*, 253-263, doi:10.5511/plantbiotechnology.19.1030a.
77. Swinnen, G.; De Meyer, M.; Pollier, J.; Molina-Hidalgo, F.J.; Ceulemans, E.; De Clercq, R.; Bossche, R.V.; Fernández-Calvo, P.; Ron, M.; Pauwels, L., et al. Constitutive steroidal glycoalkaloid biosynthesis in tomato is regulated by the clade IIIe basic helix-loop-helix transcription factors MYC1 and MYC2. *bioRxiv* **2020**, *2020.01.27.921833*, doi:10.1101/2020.01.27.921833.
78. Ueta, R.; Abe, C.; Watanabe, T.; Sugano, S.S.; Ishihara, R.; Ezura, H.; Osakabe, Y.; Osakabe, K. Rapid breeding of parthenocarpic tomato plants using CRISPR/Cas9. *Sci. Rep.* **2017**, *7*, 1-8, doi:10.1038/s41598-017-00501-4.
79. Butler, N.M.; Jansky, S.H.; Jiang, J. First-generation genome editing in potato using hairy root transformation. *Plant Biotechnol. J.* **2020**, *18*, 2201-2209, doi:10.1111/pbi.13376.
80. Čermák, T.; Curtin, S.J.; Gil-Humanes, J.; Čegan, R.; Kono, T.J.Y.; Konečná, E.; Belanto, J.J.; Starker, C.G.; Mathre, J.W.; Greenstein, R.L., et al. A multipurpose toolkit to enable advanced genome engineering in plants. *Plant Cell* **2017**, *29*, 1196-1217, doi:10.1105/tpc.16.00922.
81. Irigoyen, S.; Ramasamy, M.; Pant, S.; Niraula, P.; Bedre, R.; Gurung, M.; Rossi, D.; Laughlin, C.; Gorman, Z.; Achor, D., et al. Plant hairy roots enable high throughput identification of antimicrobials against *Candidatus* Liberibacter spp. *Nat. Commun.* **2020**, *11*, 1-14, doi:10.1038/s41467-020-19631-x.
82. Zakaria, M.M.; Schemmerling, B.; Ober, D. CRISPR/Cas9-mediated genome editing in comfrey (*Symphytum officinale*) hairy roots results in the complete eradication of pyrrolizidine alkaloids. *Molecules* **2021**, *26*, 1-22, doi:10.3390/molecules26061498.
83. Fauser, F.; Schiml, S.; Puchta, H. Both CRISPR/Cas-based nucleases and nickases can be used efficiently for genome engineering in *Arabidopsis thaliana*. *Plant J.* **2014**, *79*, 348-359, doi:10.1111/tpj.12554.
84. Iaffaldano, B.; Zhang, Y.; Cornish, K. CRISPR/Cas9 genome editing of rubber producing dandelion *Taraxacum kok-saghyz* using *Agrobacterium rhizogenes* without selection. *Ind. Crops Prod.* **2016**, *89*, 356-362, doi:10.1016/j.indcrop.2016.05.029.

85. Nekrasov, V.; Staskawicz, B.; Weigel, D.; Jones, J.D.G.; Kamoun, S. Targeted mutagenesis in the model plant *Nicotiana benthamiana* using Cas9 RNA-guided endonuclease. *Nat. Biotechnol.* **2013**, *31*, 691-693, doi:10.1038/nbt.2655.
86. Ji, J.; Zhang, C.; Sun, Z.; Wang, L.; Duanmu, D.; Fan, Q. Genome editing in cowpea *Vigna unguiculata* using CRISPR-Cas9. *Int. J. Mol. Sci.* **2019**, *20*, 1-13, doi:10.3390/ijms20102471.
87. Herrera-Estrella, L.; De Block, M.; Messens, E.; Hernalsteens, J.-P.; Van Montagu, M.; Schell, J. Chimeric genes as dominant selectable markers in plant cells. *EMBO J.* **1983**, *2*, 987-995, doi:10.1002/j.1460-2075.1983.tb01532.x.
88. Beck, E.; Ludwig, G.; Auerswald, E.A.; Reiss, B.; Schaller, H. Nucleotide sequence and exact localization of the neomycin phosphotransferase gene from transposon Tn*5*. *Gene* **1982**, *19*, 327-336, doi:10.1016/0378-1119(82)90023-3.
89. Fling, M.E.; Walton, L.; Elwell, L.P. Monitoring of plasmid-encoded, trimethoprim-resistant dihydrofolate reductase genes: detection of a new resistant enzyme. *Antimicrob. Agents Chemother.* **1982**, *22*, 882-888, doi:10.1128/AAC.22.5.882.
90. Waldron, C.; Murphy, E.B.; Roberts, J.L.; Gustafson, G.D.; Armour, S.L.; Malcolm, S.K. Resistance to hygromycin B. *A new marker for plant transformation studies. Plant Mol. Biol.* **1985**, *5*, 103-108, doi:10.1007/BF00020092.
91. Rao, R.N.; Allen, N.E.; Hobbs, J.N., Jr.; Alborn, W.E., Jr.; Kirst, H.A.; Paschal, J.W. Genetic and enzymatic basis of hygromycin B resistance in *Escherichia coli*. *Antimicrob. Agents Chemother.* **1983**, *24*, 689-695, doi:10.1128/AAC.24.5.689.
92. Gritz, L.; Davies, J. Plasmid-encoded hygromycin B resistance: the sequence of hygromycin B phosphotransferase gene and its expression in *Escherichia coli* and *Saccharomyces cerevisiae*. *Gene* **1983**, *25*, 179-188, doi:10.1016/0378-1119(83)90223-8.
93. Comai, L.; Facciotti, D.; Hiatt, W.R.; Thompson, G.; Rose, R.E.; Stalker, D.M. Expression in plants of a mutant *aroA* gene from *Salmonella typhimurium* confers tolerance to glyphosate. *Nature* **1985**, *317*, 741-744, doi:10.1038/317741a0.
94. Comai, L.; Sen, L.C.; Stalker, D.M. An altered *aroA* gene product confers resistance to the herbicide glyphosate. *Science* **1983**, *221*, 370-373, doi:10.1126/science.221.4608.370.
95. Hille, J.; Verheggen, F.; Roelvink, P.; Franssen, H.; van Kammen, A.; Zabel, P. Bleomycin resistance: a new dominant selectable marker for plant cell transformation. *Plant Mol. Biol.* **1986**, *7*, 171-176, doi:10.1007/BF00021328.
96. Collis, C.M.; Hall, R.M. Identification of a Tn*5* determinant conferring resistance to phleomycins, bleomycins, and tallysomycins. *Plasmid* **1985**, *14*, 143-151, doi:10.1016/0147-619X(85)90074-5.
97. Mazodier, P.; Cossart, P.; Giraud, E.; Gasser, F. Completion of the nucleotide sequence of the central region of Tn*5* confirms the presence of three resistance genes. *Nucleic Acids Res.* **1985**, *13*, 195-205, doi:10.1093/nar/13.1.195.
98. De Block, M.; Botterman, J.; Vandewiele, M.; Dockx, J.; Thoen, C.; Gosselé, V.; Movva, N.R.; Thompson, C.; Van Montagu, M.; Leemans, J. Engineering herbicide resistance in plants by expression of a detoxifying enzyme. *EMBO J.* **1987**, *6*, 2513-2518, doi:10.1002/j.1460-2075.1987.tb02537.x.

99. Murakami, T.; Anzai, H.; Imai, S.; Satoh, A.; Nagaoka, K.; Thompson, C.J. The bialaphos biosynthetic genes of *Streptomyces hygroscopicus*: molecular cloning and characterization of the gene cluster. *Mol. Gen. Genet.* **1986**, *205*, 42-53, doi:10.1007/BF02428031.
100. Jones, J.D.G.; Svab, Z.; Harper, E.C.; Hurwitz, C.D.; Maliga, P. A dominant nuclear streptomycin resistance marker for plant cell transformation. *Mol. Gen. Genet.* **1987**, *210*, 86-91, doi:10.1007/BF00337762.
101. Mazodier, P.; Giraud, E.; Gasser, F. Genetic analysis of the streptomycin resistance encoded by Tn*5*. *Mol. Gen. Genet.* **1983**, *192*, 155-162, doi:10.1007/BF00327661.
102. Haughn, G.W.; Smith, J.; Mazur, B.; Somerville, C. Transformation with a mutant *Arabidopsis* acetolactate synthase gene renders tobacco resistant to sulfonylurea herbicides. *Mol. Gen. Genet.* **1988**, *211*, 266-271, doi:10.1007/BF00330603.
103. Hayford, M.B.; Medford, J.I.; Hoffman, N.L.; Rogers, S.G.; Klee, H.J. Development of a plant transformation selection system based on expression of genes encoding gentamicin acetyltransferases. *Plant Physiol.* **1988**, *86*, 1216-1222, doi:10.1104/pp.86.4.1216.
104. Bräu, B.; Pilz, U.; Piepersberg, W. Genes for gentamicin-(3)-*N*-acetyltransferases III and IV: I. Nucleotide sequence of the *AAC(3)-IV* gene and possible involvement of an IS*140* element in its expression. *Mol. Gen. Genet.* **1984**, *193*, 179-187, doi:10.1007/BF00327434.
105. Allmansberger, R.; Bräu, B.; Piepersberg, W. Genes for gentamicin-(3)-*N*-acetyltransferases III and IV: II. Nucleotide sequences of three *AAC(3)-III* genes and evolutionary aspects. *Mol. Gen. Genet.* **1985**, *198*, 514-520, doi:10.1007/BF00332949.
106. Klein, T.M.; Gradziel, T.; Fromm, M.E.; Sanford, J.C. Factors influencing gene delivery into *Zea mays* cells by high–velocity microprojectiles. *Bio/Technology* **1988**, *6*, 559-563, doi:10.1038/nbt0588-559.
107. Novel, G.; Novel, M. Mutants d'*Escherichia coli* K 12 affectés pour leur croissance sur méthyl-*β*-D-glucuronide: localisation du gène de structure de la *β*-D-glucuronidase (*uid A*). *Mol. Gen. Genet.* **1973**, *120*, 319-335, doi:10.1007/BF00268146.
108. Jefferson, R.A.; Burgess, S.M.; Hirsh, D. *β*-Glucuronidase from *Escherichia coli* as a gene-fusion marker. *Proc. Natl. Acad. Sci. U.S.A.* **1986**, *83*, 8447-8451, doi:10.1073/pnas.83.22.8447.
109. Streber, W.R.; Willmitzer, L. Transgenic tobacco plants expressing a bacterial detoxifying enzyme are resistant to 2,4-D. *Bio/Technology* **1989**, *7*, 811-816, doi:10.1038/nbt0889-811.
110. Streber, W.R.; Timmis, K.N.; Zenk, M.H. Analysis, cloning, and high-level expression of 2,4-dichlorophenoxyacetate monooxygenase gene *tfdA* of *Alcaligenes eutrophus* JMP134. *J. Bacteriol.* **1987**, *169*, 2950-2955, doi:10.1128/jb.169.7.2950-2955.1987.
111. Perez, P.; Tiraby, G.; Kallerhoff, J.; Perret, J. Phleomycin resistance as a dominant selectable marker for plant cell transformation. *Plant Mol. Biol.* **1989**, *13*, 365-373, doi:10.1007/BF00015548.
112. Drocourt, D.; Calmels, T.; Reynes, J.-P.; Baron, M.; Tiraby, G. Cassettes of the *Streptoalloteichus hindustanus ble* gene for transformation of lower and higher eukaryotes to phleomycin resistance. *Nucleic Acids Res.* **1990**, *18*, 4009, doi:10.1093/nar/18.13.4009.

113. Ludwig, S.R.; Bowen, B.; Beach, L.; Wessler, S.R. A regulatory gene as a novel visible marker for maize transformation. *Science* **1990**, *247*, 449–450, doi:10.1126/science.247.4941.449

114. Ludwig, S.R.; Habera, L.F.; Dellaporta, S.L.; Wessler, S.R. *Lc*, a member of the maize *R* gene family responsible for tissue-specific anthocyanin production, encodes a protein similar to transcriptional activators and contains the *myc*-homology region. *Proc. Natl. Acad. Sci. U.S.A.* **1989**, *86*, 7092-7096, doi:10.1073/pnas.86.18.7092.

115. Svab, Z.; Harper, E.C.; Jones, J.D.G.; Maliga, P. Aminoglycoside-3"-adenyltransferase confers resistance to spectinomycin and streptomycin in *Nicotiana tabacum*. *Plant Mol. Biol.* **1990**, *14*, 197-205, doi:10.1007/BF00018560.

116. Hollingshead, S.; Vapnek, D. Nucleotide sequence analysis of a gene encoding a streptomycin/spectinomycin adenyltransferase. *Plasmid* **1985**, *13*, 17-30, doi:10.1016/0147-619X(85)90052-6.

117. Kamakura, T.; Yoneyama, K.; Yamaguchi, I. Expression of the blasticidin S deaminase gene (*bsr*) in tobacco: fungicide tolerance and a new selective marker for transgenic plants. *Mol. Gen. Genet.* **1990**, *223*, 332-334, doi:10.1007/BF00265072.

118. Endo, T.; Furuta, K.; Kaneko, A.; Katsuki, T.; Kobayashi, K.; Azuma, A.; Watanabe, A.; Shimazu, A. Inactivation of blasticidin S by *Bacillus cereus*. I. Inactivation mechanism. *J. Antibiot.* **1987**, *40*, 1791-1793, doi:10.7164/antibiotics.40.1791.

119. Endo, T.; Kobayashi, K.; Nakayama, N.; Tanaka, T.; Kamakura, T.; Yamaguchi, I. Inactivation of blasticidin S by *Bacillus cereus*. II. Isolation and characterization of a plasmid, pBSR8, from *Bacillus cereus*. *J. Antibiot.* **1988**, *41*, 271-273, doi:10.7164/antibiotics.41.271.

120. Carrer, H.; Staub, J.M.; Maliga, P. Gentamycin resistance in *Nicotiana* conferred by AAC(3)-I, a narrow substrate specificity acetyltransferase. *Plant Mol. Biol.* **1991**, *17*, 301-303, doi:10.1007/BF00039510.

121. Wohlleben, W.; Arnold, W.; Bissonnette, L.; Pelletier, A.; Tanguay, A.; Roy, P.H.; Gamboa, G.C.; Barry, G.F.; Aubert, E.; Davies, J., et al. On the evolution of Tn*21*-like multiresistance transposons: sequence analysis of the gene (*aacC1*) for gentamicin acetyltransferase-3-I(AAC(3)-I), another member of the Tn*21*-based expression cassette. *Mol. Gen. Genet.* **1989**, *217*, 202-208, doi:10.1007/BF02464882.

122. Niedz, R.P.; Sussman, M.R.; Satterlee, J.S. Green fluorescent protein: an *in vivo* reporter of plant gene expression. *Plant Cell Rep.* **1995**, *14*, 403-406, doi:10.1007/BF00234043.

123. Prasher, D.C.; Eckenrode, V.K.; Ward, W.W.; Prendergast, F.G.; Cormier, M.J. Primary structure of the *Aequorea victoria* green-fluorescent protein. *Gene* **1992**, *111*, 229-233, doi:10.1016/0378-1119(92)90691-H.

124. Joersbo, M.; Donaldson, I.; Kreiberg, J.; Petersen, S.G.; Brunstedt, J.; Okkels, F.T. Analysis of mannose selection used for transformation of sugar beet. *Mol. Breed.* **1998**, *4*, 111-117, doi:10.1023/A:1009633809610.

125. Miles, J.S.; Guest, J.R. Nucleotide sequence and transcriptional start point of the phosphomannose isomerase gene (*manA*) of *Escherichia coli*. *Gene* **1984**, *32*, 41-48, doi:10.1016/0378-1119(84)90030-1.

126. Haldrup, A.; Petersen, S.G.; Okkels, F.T. The xylose isomerase gene from *Thermoanaerobacterium thermosulfurogenes* allows effective selection of transgenic plant cells using D-xylose as the selection agent. *Plant Mol. Biol.* **1998**, *37*, 287-296, doi:10.1023/A:1005910417789.

127. Lee, C.Y.; Bagdasarian, M.; Meng, M.H.; Zeikus, J.G. Catalytic mechanism of xylose (glucose) isomerase from *Clostridium thermosulfurogenes*. Characterization of the structural gene and function of active site histidine. *J. Biol. Chem.* **1990**, *265*, 19082-19090, doi:10.1016/S0021-9258(17)30628-2.
128. Jach, G.; Binot, E.; Frings, S.; Luxa, K.; Schell, J. Use of red fluorescent protein from *Discosoma* sp. (dsRED) as a reporter for plant gene expression. *Plant J.* **2001**, *28*, 483-491, doi:10.1046/j.1365-313X.2001.01153.x.
129. Matz, M.V.; Fradkov, A.F.; Labas, Y.A.; Savitsky, A.P.; Zaraisky, A.G.; Markelov, M.L.; Lukyanov, S.A. Fluorescent proteins from nonbioluminescent Anthozoa species. *Nat. Biotechnol.* **1999**, *17*, 969-973, doi:10.1038/13657.
130. Leyman, B.; Avonce, N.; Ramon, M.; Van Dijck, P.; Thevelein, J.M.; Iturriaga, G. New selection marker for plant transformation. In *Recombinant Gene Expression. Reviews and Protocols*, 2nd ed.; Balbás, P., Lorence, A., Eds. Humana Press: Totowa, New Jersey, USA, 2004; pp. 385-396, doi:10.1385/1-59259-774-2:385.
131. Blázquez, M.A.; Santos, E.; Flores, C.-l.; Martínez-Zapater, J.M.; Salinas, J.; Gancedo, C. Isolation and molecular characterization of the *Arabidopsis TPS1* gene, encoding trehalose-6-phosphate synthase. *Plant J.* **1998**, *13*, 685-689, doi:10.1046/j.1365-313X.1998.00063.x.
